# Supplementary material for: Ball-Milling Preparation of the Drug–Drug Solid Form of Pioglitazone-Rosuvastatin at Different Molar Ratios: Characterization and Intrinsic Dissolution Rates Evaluation
Source: Pharmaceutics. 2023 Feb 13;15(2):630. doi: 10.3390/pharmaceutics15020630 (PMC9959797; doi:10.3390/pharmaceutics15020630)
Supplement: Supplementary file 1 [file pharmaceutics-15-00630-s001.zip › pharmaceutics-2161498-supplementary.pdf]

pharmaceutics

MDPI

Supplementary Materials

Ball-Milling Preparation of the Drug–Drug Solid form of Pioglitazone-Rosuvastatin at Different Molar Ratios: Characterization and Intrinsic Dissolution Rates Evaluation

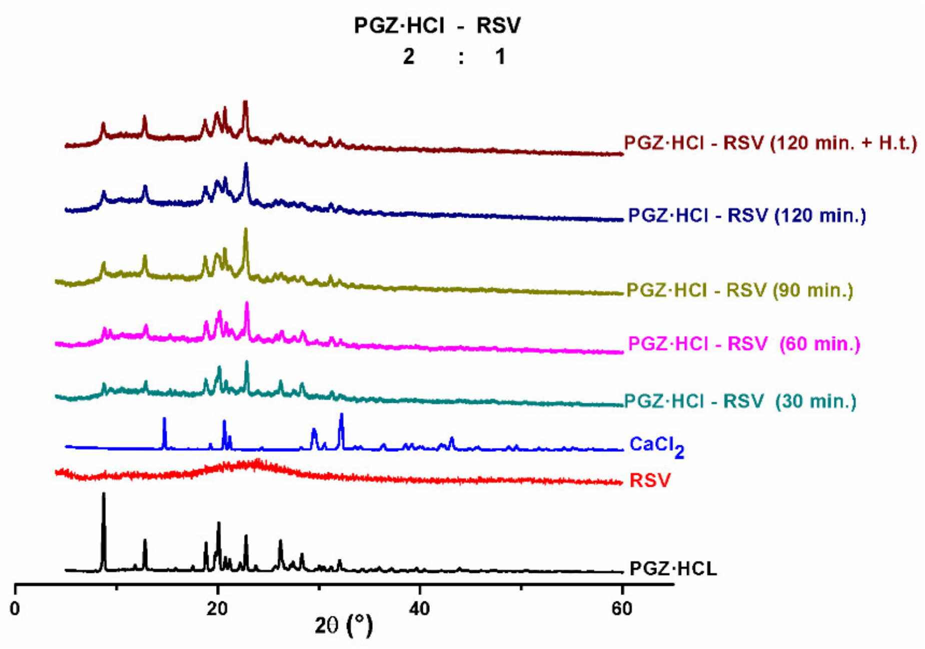

Figure S1. Diffractograms of the solid phase PGZ·HCl-RSV (2:1, EtOH) extending the reaction times.

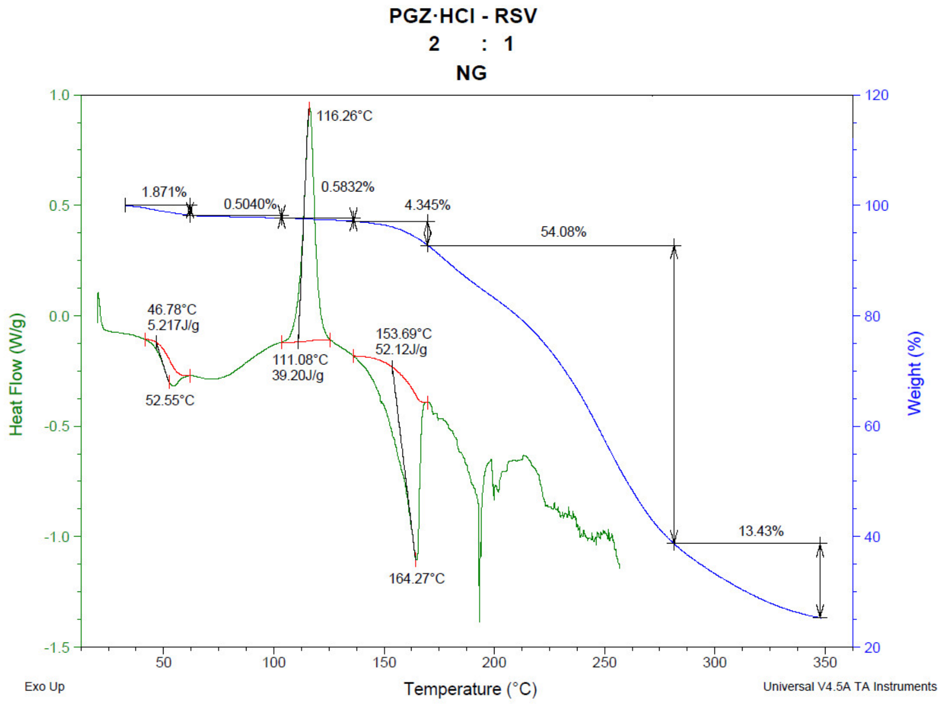

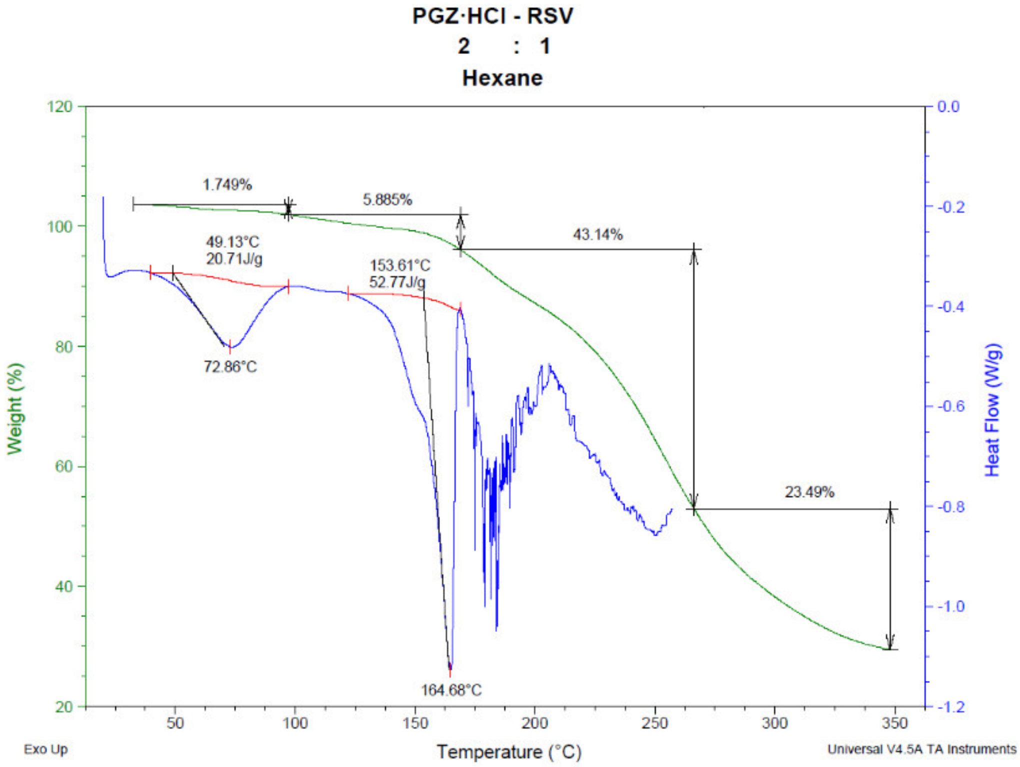
PGZ·HCl - RSV
2 : 1
Hexane
1.749%
5.885%
43.14%
23.49%
49.13°C
20.71J/g
72.86°C
153.61°C
52.77J/g
164.68°C
Weight (%)
Heat Flow (W/g)
Temperature (°C)
Exo Up
Universal V4.5A TA Instruments

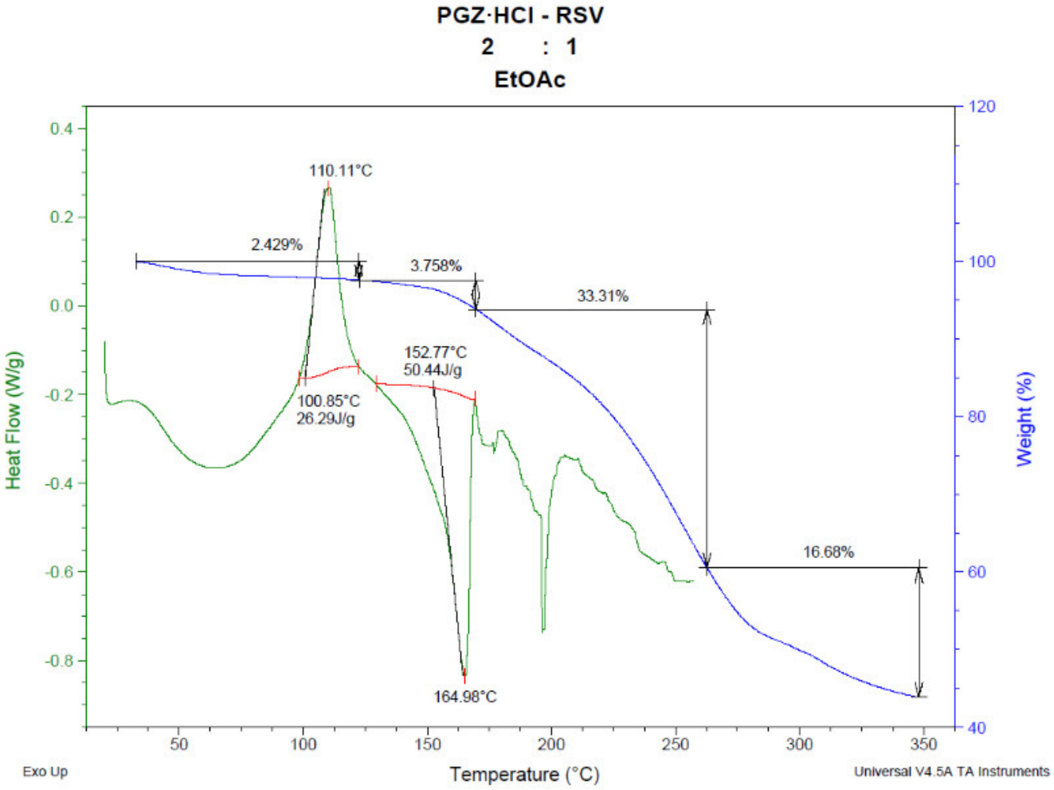
PGZ·HCl - RSV
2 : 1
EtOAc
110.11°C
2.429%
3.758%
33.31%
16.68%
100.85°C
26.29J/g
152.77°C
50.44J/g
164.98°C
Heat Flow (W/g)
Weight (%)
Temperature (°C)
Exo Up
Universal V4.5A TA Instruments

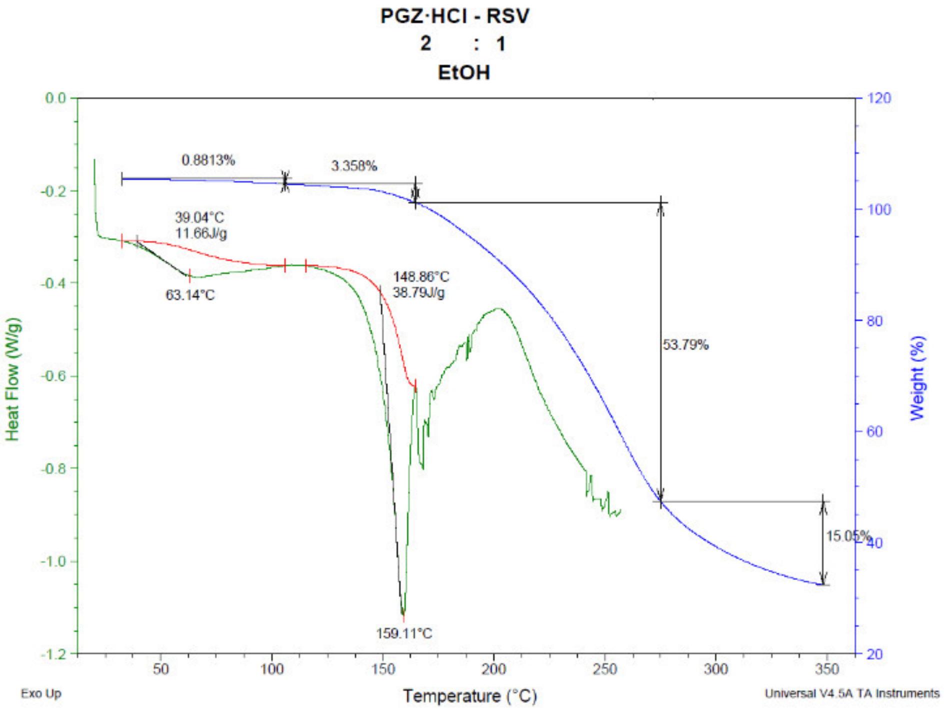
PGZ·HCl - RSV
2 : 1
EtOH
0.8813%
3.358%
39.04°C
11.66J/g
63.14°C
148.86°C
38.79J/g
53.79%
15.05%
159.11°C
Heat Flow (W/g)
Weight (%)
Temperature (°C)
Exo Up
Universal V4.5A TA Instruments

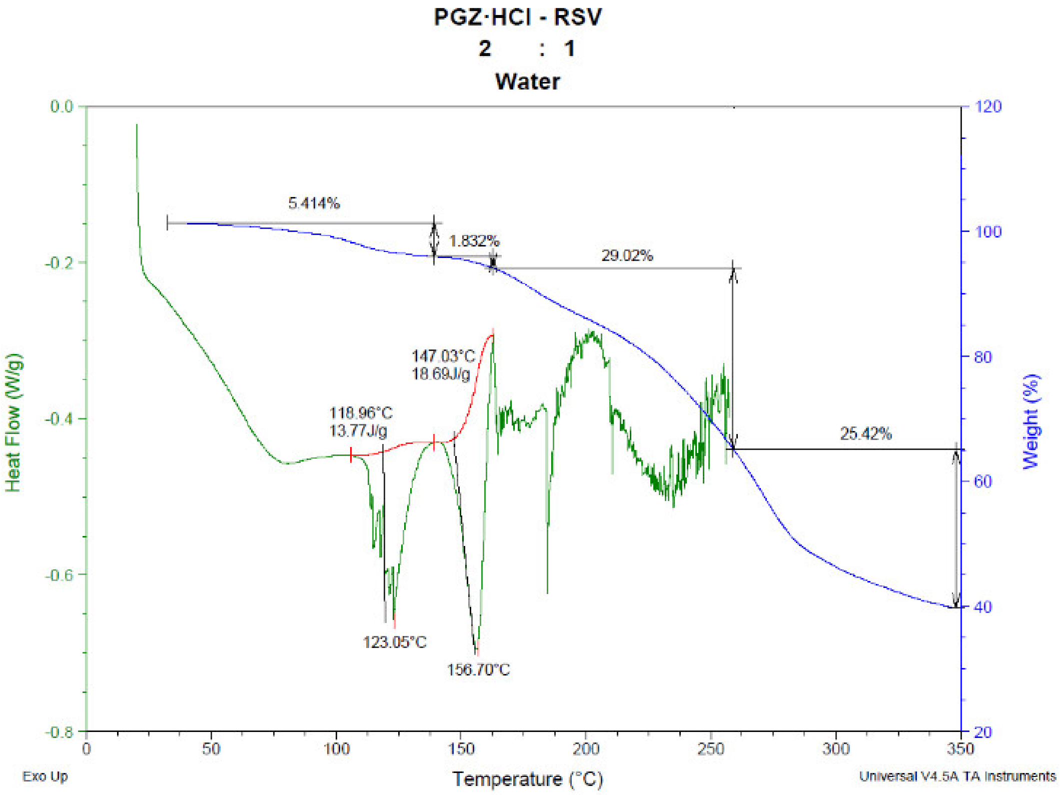
PGZ·HCl - RSV
2 : 1
Water
5.414%
1.832%
29.02%
147.03°C
18.69J/g
118.96°C
13.77J/g
25.42%
123.05°C
156.70°C
Heat Flow (W/g)
Weight (%)
Temperature (°C)
Exo Up
Universal V4.5A TA Instruments

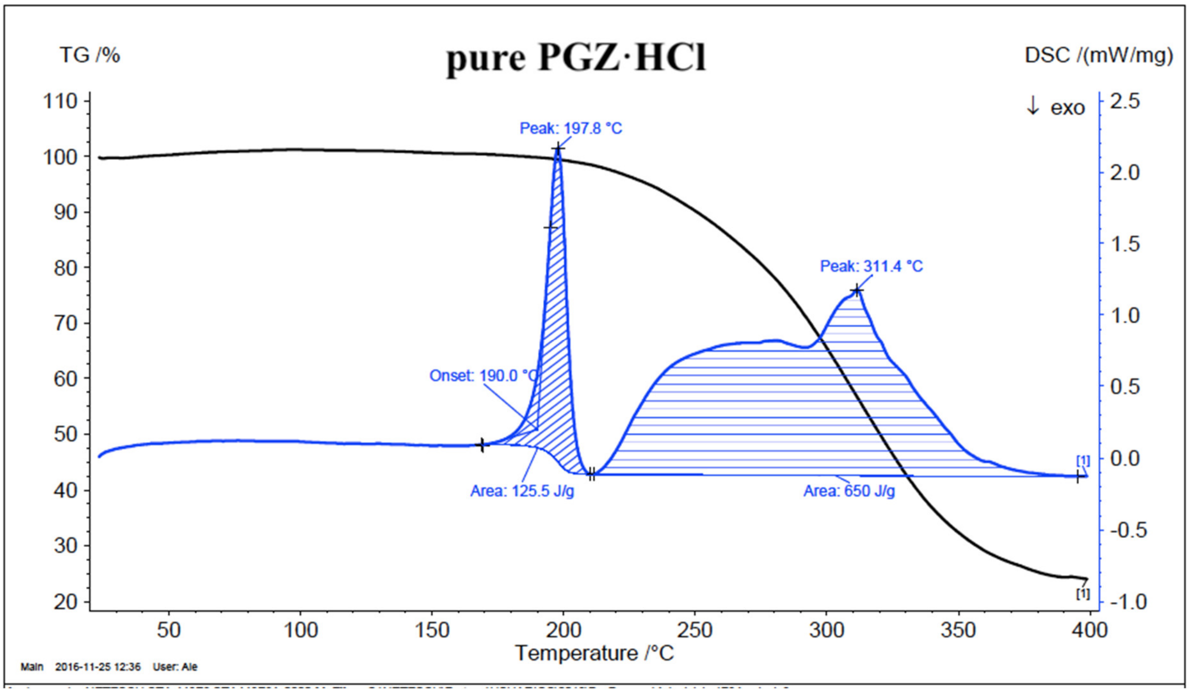
pure PGZ·HCl
TG /%
DSC /(mW/mg)
↓ exo
Peak: 197.8 °C
Peak: 311.4 °C
Onset: 190.0 °C
Area: 125.5 J/g
Area: 650 J/g
[1]
Temperature /°C
Main 2016-11-25 12:36 User: Ale

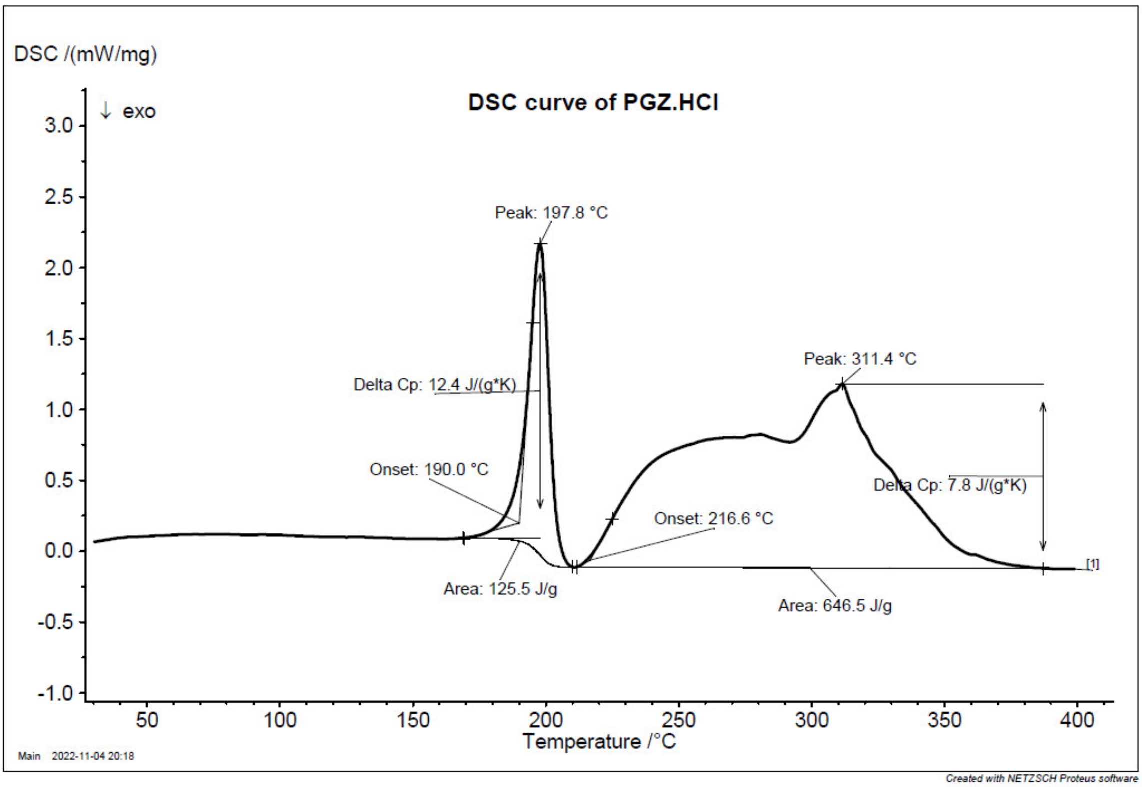
DSC /(mW/mg)
↓ exo
DSC curve of PGZ.HCl
Peak: 197.8 °C
Delta Cp: 12.4 J/(g*K)
Onset: 190.0 °C
Area: 125.5 J/g
Peak: 311.4 °C
Delta Cp: 7.8 J/(g*K)
Onset: 216.6 °C
Area: 646.5 J/g
[1]
Temperature /°C
Main 2022-11-04 20:18
Created with NETZSCH Proteus software

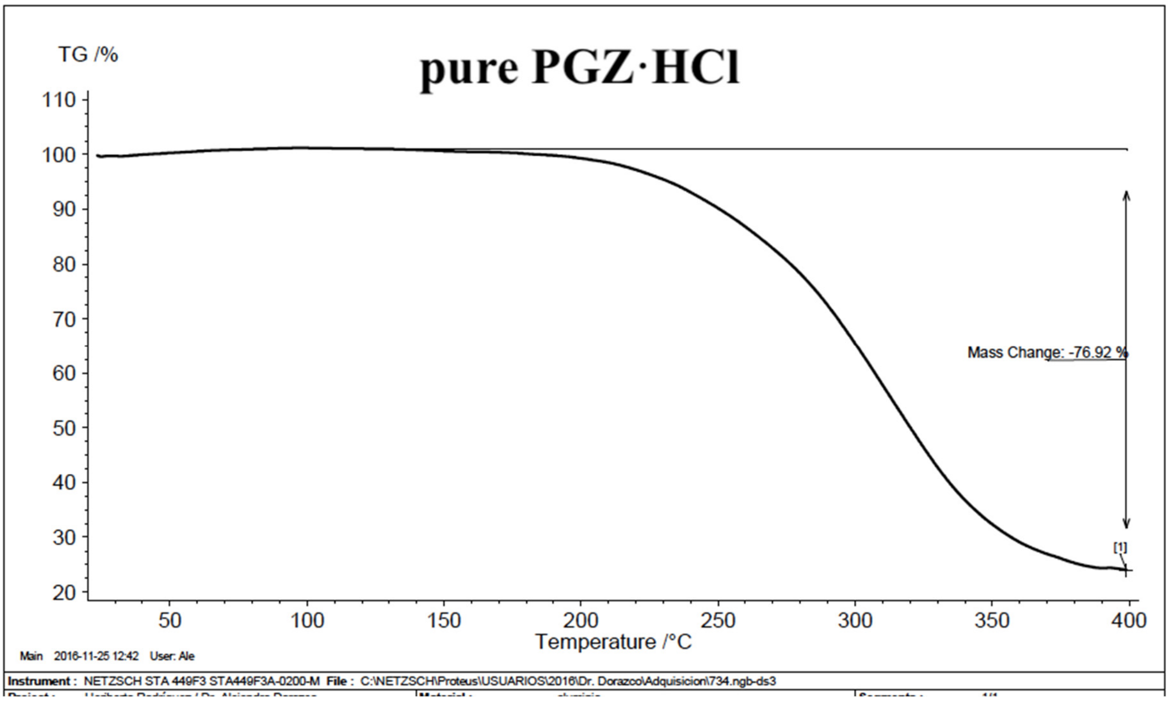
pure PGZ·HCl
TG /%
Mass Change: -76.92 %
[1]
Temperature /°C
Main 2016-11-25 12:42 User: Ale
Instrument : NETZSCH STA 449F3 STA449F3A-0200-M File : C:\NETZSCH\Proteus\USUARIOS\2016\Dr. Dorazco\Adquisicion\734.ngb-ds3

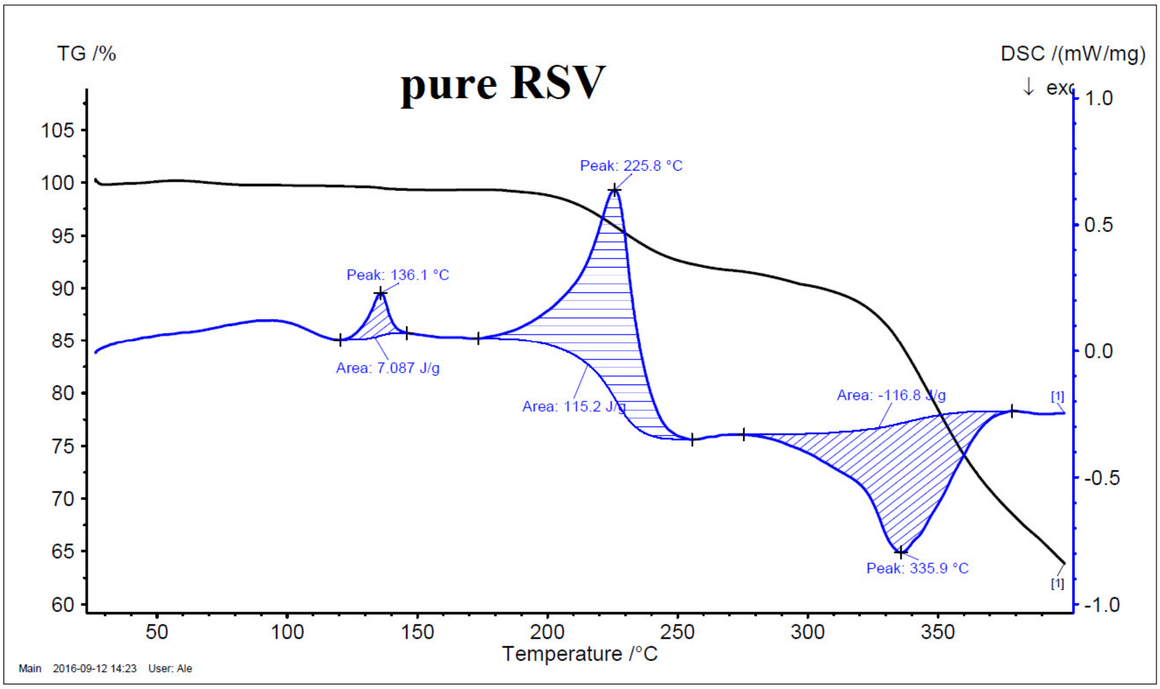
pure RSV
TG /%
DSC /(mW/mg)
↓ exo
Peak: 225.8 °C
Peak: 136.1 °C
Area: 7.087 J/g
Area: 115.2 J/g
Area: -116.8 J/g
Peak: 335.9 °C
[1]
Temperature /°C
Main 2016-09-12 14:23 User: Ale

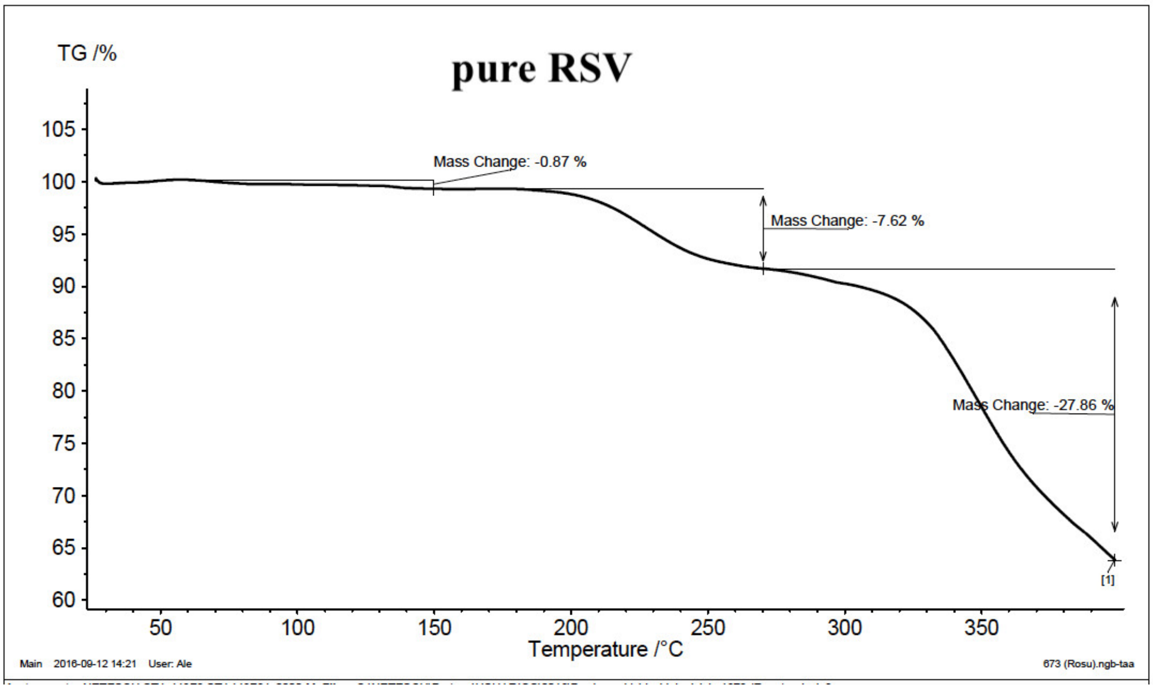
pure RSV
TG /%
Mass Change: -0.87 %
Mass Change: -7.62 %
Mass Change: -27.86 %
105
100
95
90
85
80
75
70
65
60
50
100
150
200
250
300
350
Temperature /°C
[1]
Main 2016-09-12 14:21 User: Ale
673 (Rosu).ngb-taa

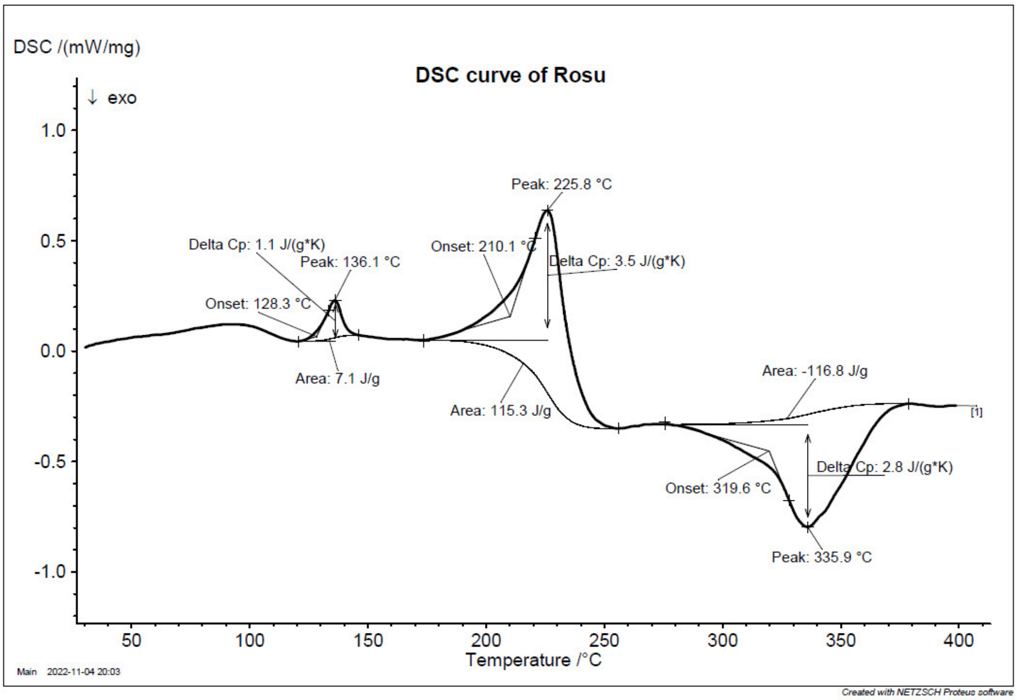
DSC /(mW/mg)
DSC curve of Rosu
↓ exo
1.0
0.5
0.0
-0.5
-1.0
Peak: 225.8 °C
Delta Cp: 1.1 J/(g*K)
Peak: 136.1 °C
Onset: 210.1 °C
Delta Cp: 3.5 J/(g*K)
Onset: 128.3 °C
Area: 7.1 J/g
Area: 115.3 J/g
Area: -116.8 J/g
[1]
Delta Cp: 2.8 J/(g*K)
Onset: 319.6 °C
Peak: 335.9 °C
50
100
150
200
250
300
350
400
Temperature /°C
Main 2022-11-04 20:03
Created with NETZSCH Proteus software

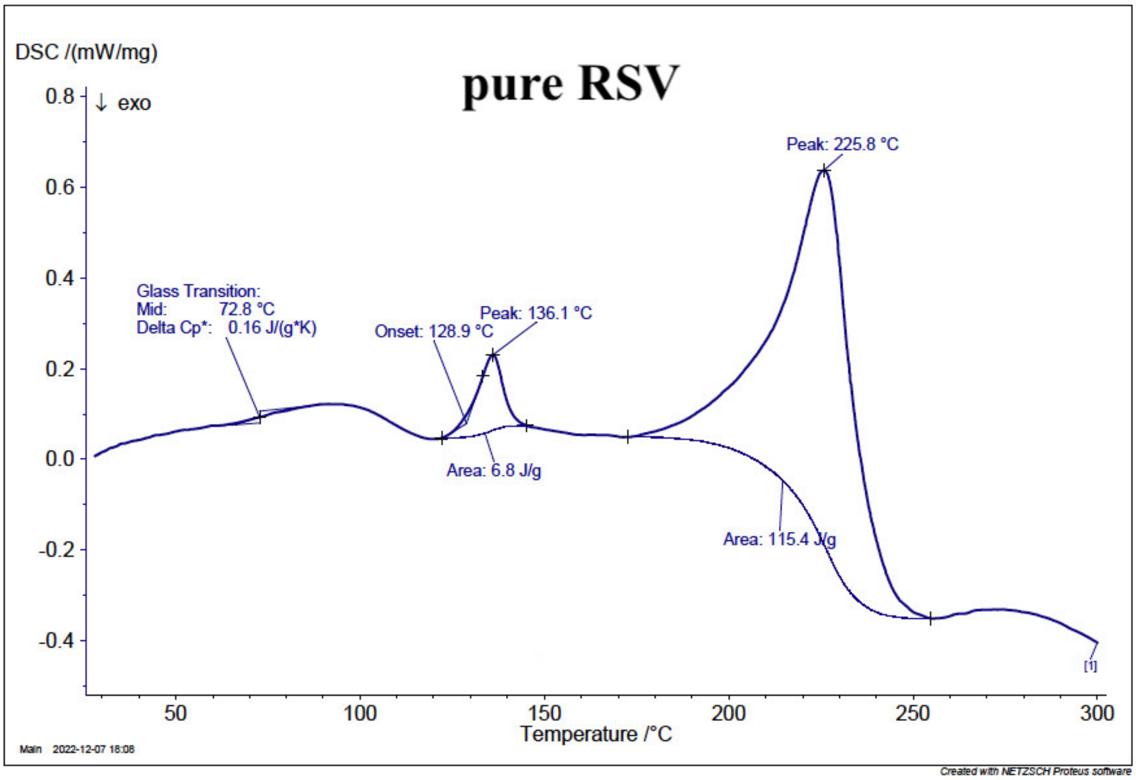

Figure S2. Individual DSC-TGA thermograms of the solid phases PGZ·HCl-RSV (2:1) prepared by NG or LAG solvent-screening. Also, individual DSC-TGA thermograms of pure PGZ·HCl and RSV.

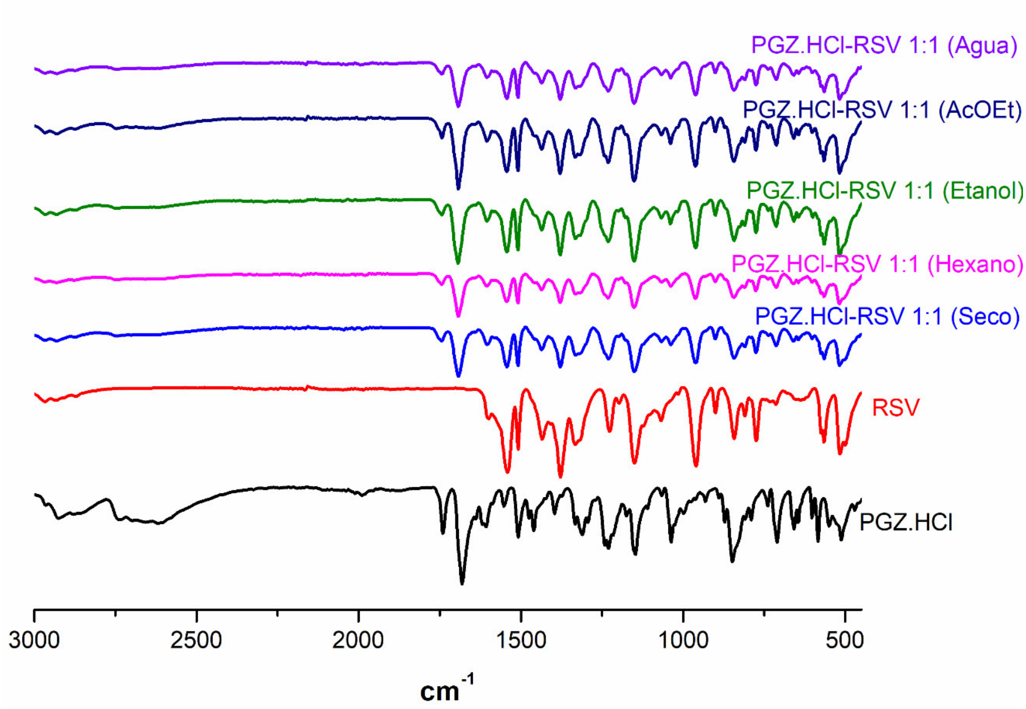

Figure S3. Full FT-IR spectra of the solid forms of PGZ·HCl-FLV (2:1) by NG or LAG solvent-screening

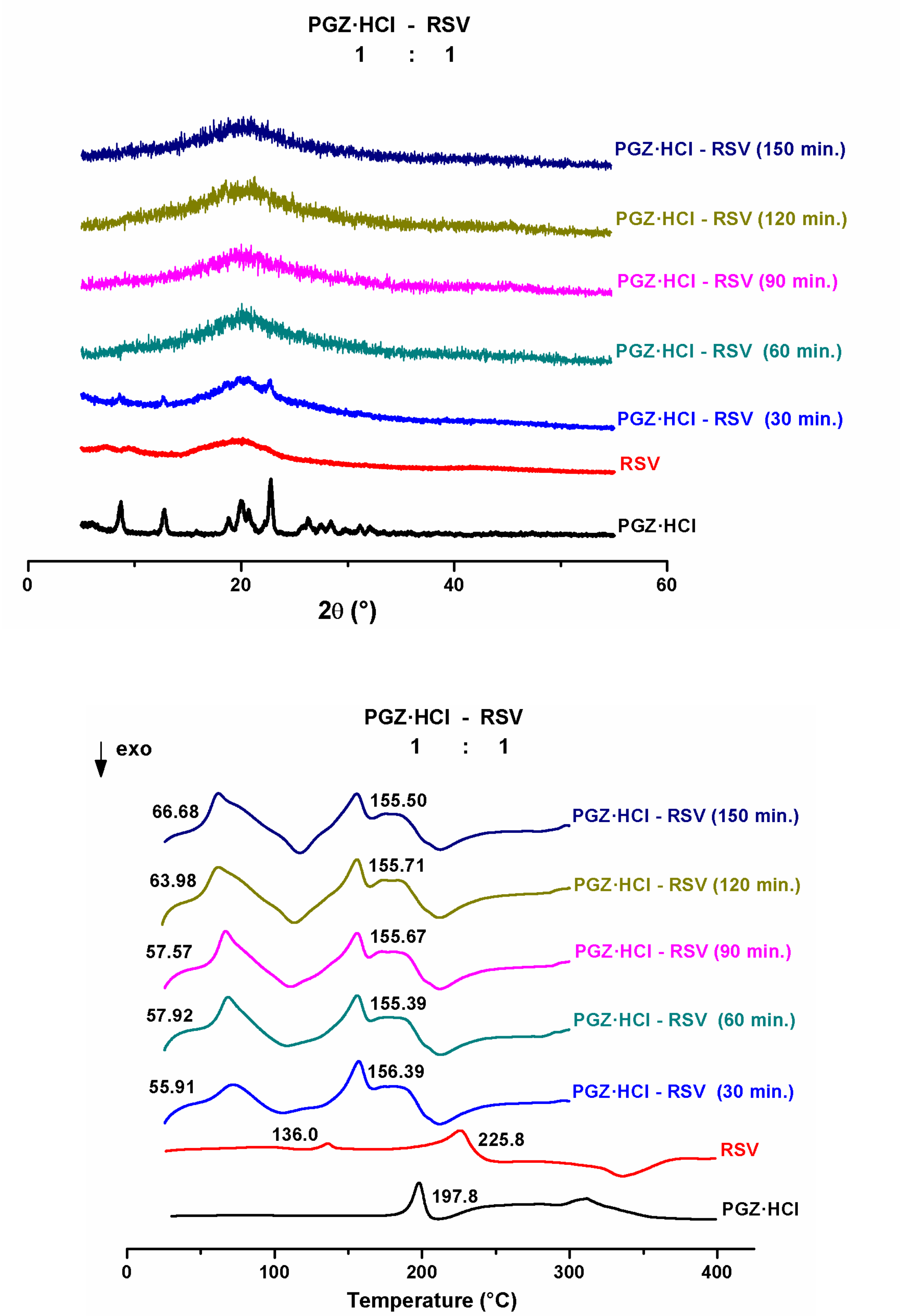

Figure S4. Grinding of the solid phase PGZ·HCl-RSV (1:1) at different times (30, 60, 90, 120, and 150 min). DSC-TGA and PXRD results.

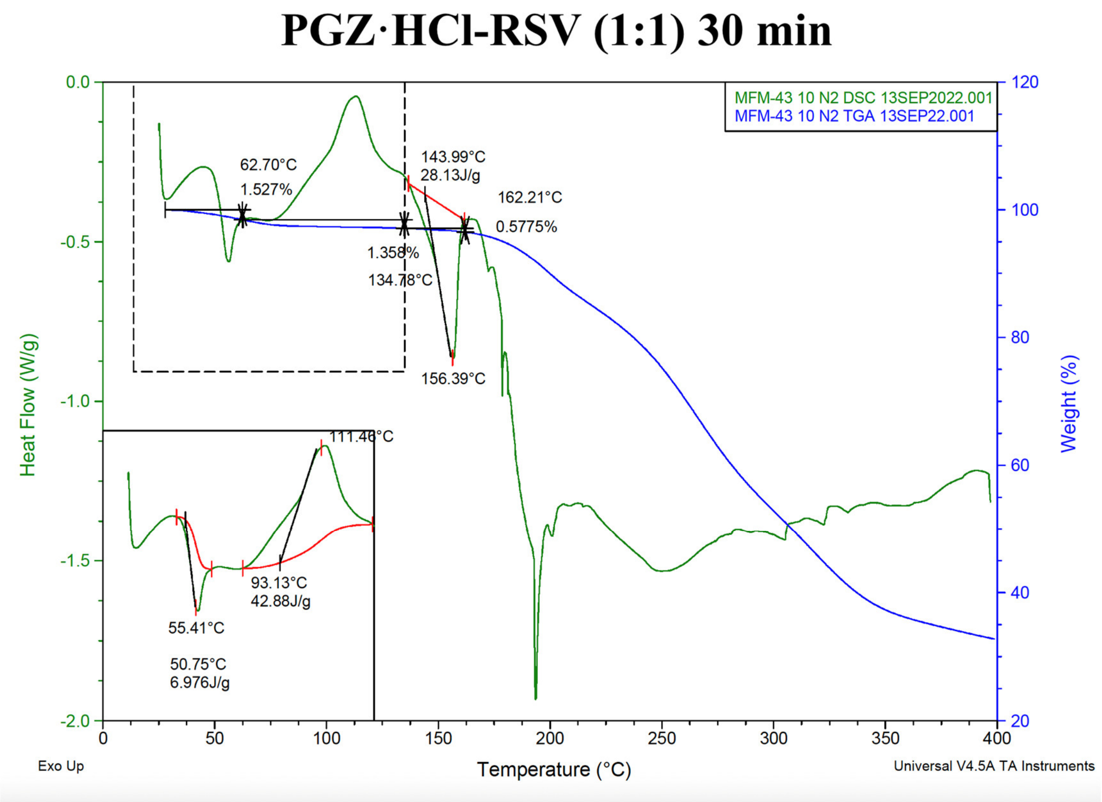
PGZ·HCl-RSV (1:1) 30 min
MFM-43 10 N2 DSC 13SEP2022.001
MFM-43 10 N2 TGA 13SEP22.001
62.70°C
1.527%
143.99°C
28.13J/g
162.21°C
0.5775%
1.358%
134.78°C
156.39°C
111.46°C
93.13°C
42.88J/g
55.41°C
50.75°C
6.976J/g
Heat Flow (W/g)
Weight (%)
Temperature (°C)
Exo Up
Universal V4.5A TA Instruments

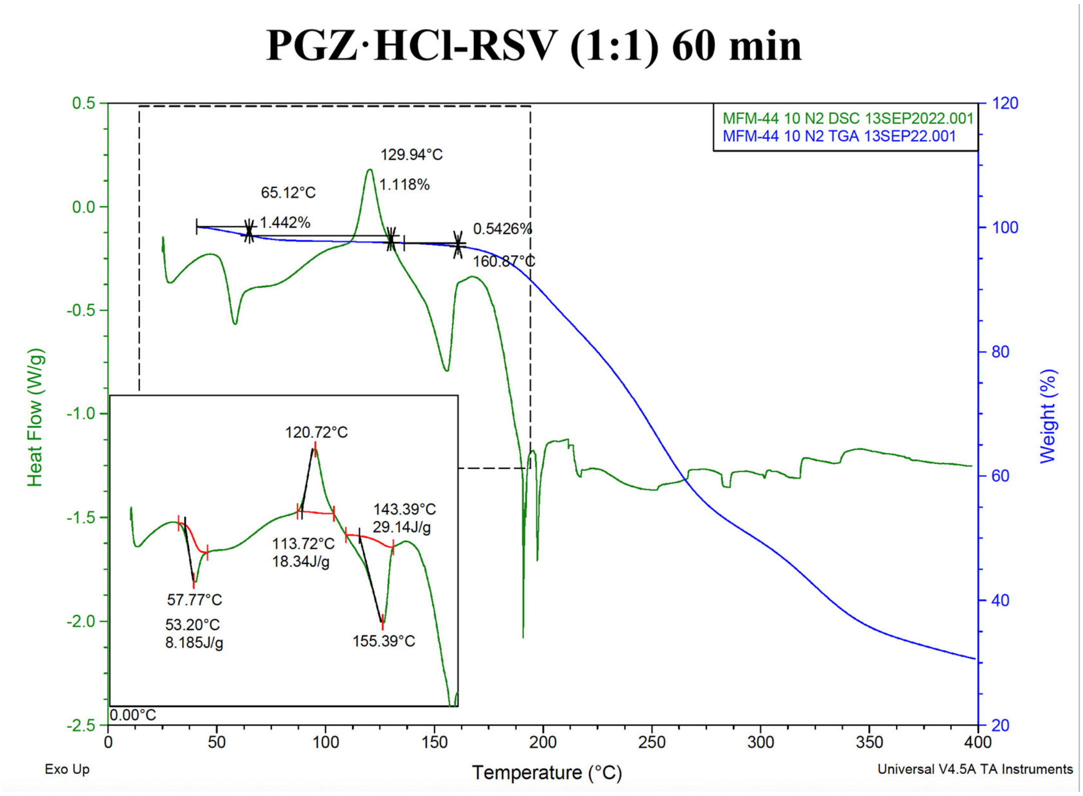
PGZ·HCl-RSV (1:1) 60 min
MFM-44 10 N2 DSC 13SEP2022.001
MFM-44 10 N2 TGA 13SEP22.001
129.94°C
1.118%
65.12°C
1.442%
0.5426%
160.87°C
120.72°C
143.39°C
29.14J/g
113.72°C
18.34J/g
57.77°C
53.20°C
8.185J/g
155.39°C
0.00°C
Heat Flow (W/g)
Weight (%)
Temperature (°C)
Exo Up
Universal V4.5A TA Instruments

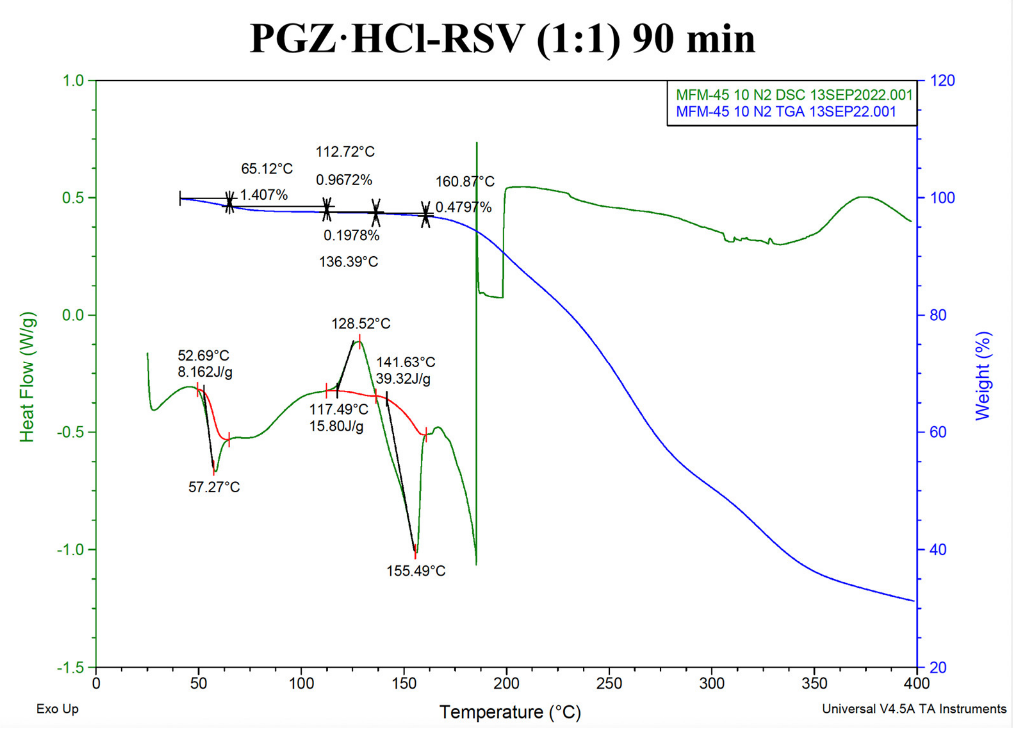
PGZ·HCl-RSV (1:1) 90 min
MFM-45 10 N2 DSC 13SEP2022.001
MFM-45 10 N2 TGA 13SEP22.001
65.12°C
1.407%
112.72°C
0.9672%
0.1978%
136.39°C
160.87°C
0.4797%
52.69°C
8.162J/g
57.27°C
128.52°C
117.49°C
15.80J/g
141.63°C
39.32J/g
155.49°C
Heat Flow (W/g)
Weight (%)
Temperature (°C)
Exo Up
Universal V4.5A TA Instruments

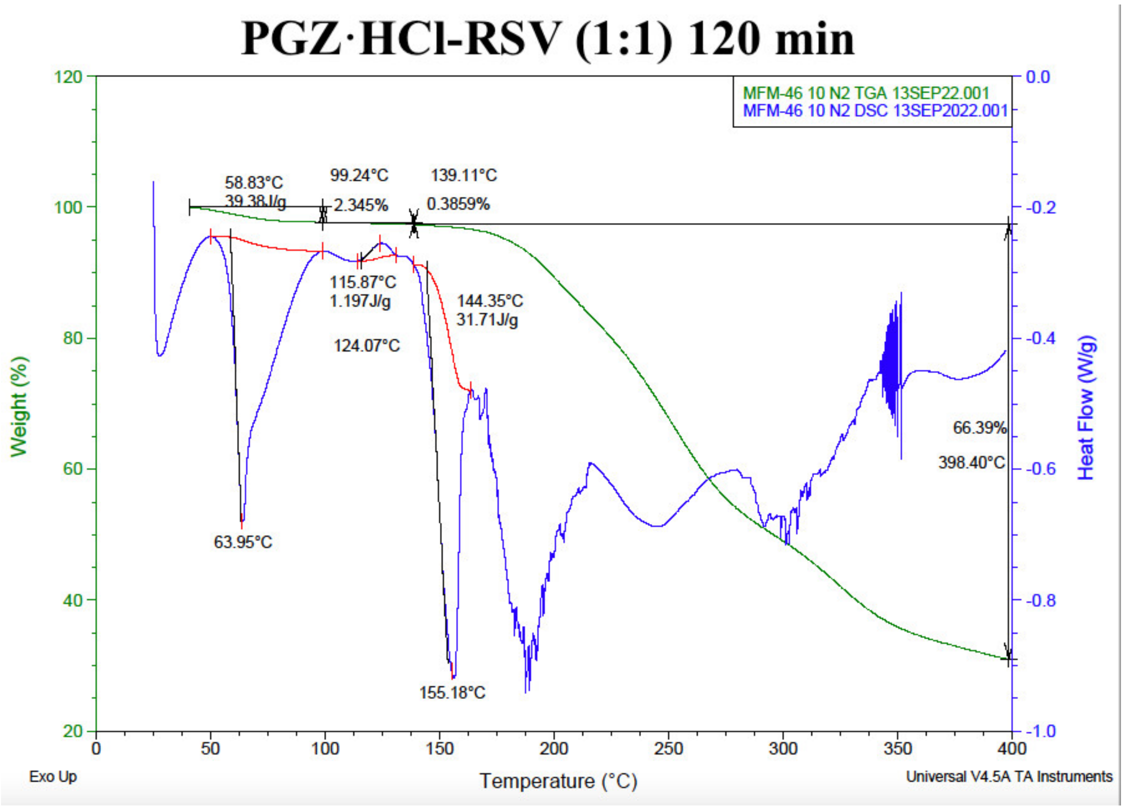
PGZ·HCl-RSV (1:1) 120 min
MFM-46 10 N2 TGA 13SEP22.001
MFM-46 10 N2 DSC 13SEP2022.001
58.83°C
39.38J/g
99.24°C
2.345%
139.11°C
0.3859%
115.87°C
1.197J/g
124.07°C
144.35°C
31.71J/g
63.95°C
155.18°C
66.39%
398.40°C
Weight (%)
Heat Flow (W/g)
Temperature (°C)
Exo Up
Universal V4.5A TA Instruments

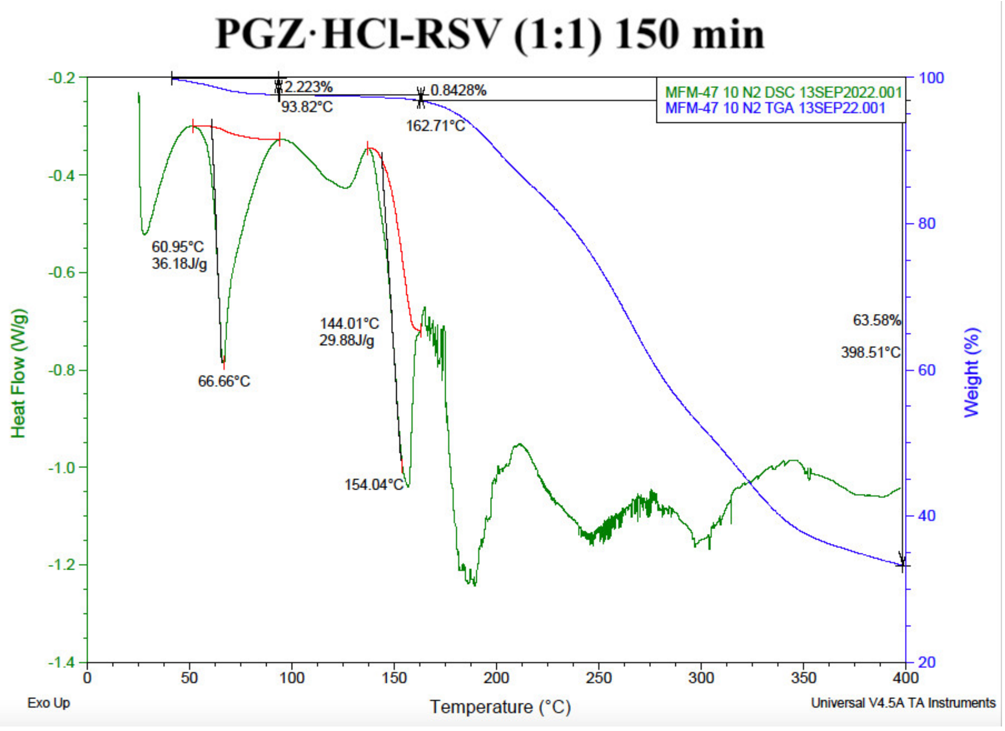

Figure S5. Individual DSC thermograms of the solid phase PGZ·HCl-RSV (1:1) at different grinding times (30, 60, 90, 120, and 150 min).

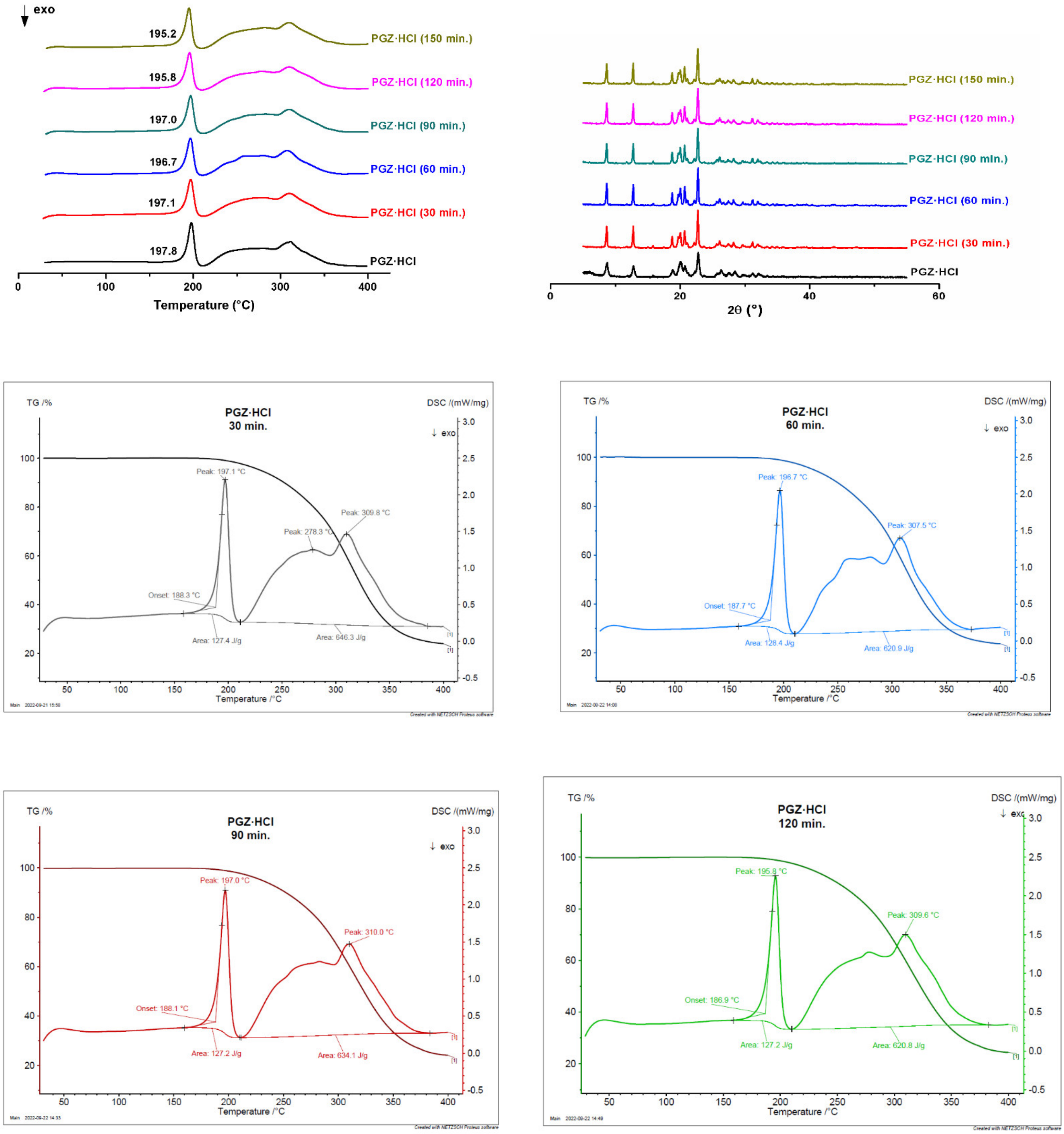
exo
195.2
195.8
197.0
196.7
197.1
197.8
PGZ·HCl (150 min.)
PGZ·HCl (120 min.)
PGZ·HCl (90 min.)
PGZ·HCl (60 min.)
PGZ·HCl (30 min.)
PGZ·HCl
Temperature (°C)
2θ (°)
TG /%
DSC /(mW/mg)
PGZ·HCl
30 min.
Peak: 197.1 °C
Peak: 278.3 °C
Peak: 309.8 °C
Onset: 188.3 °C
Area: 127.4 J/g
Area: 646.3 J/g
60 min.
Peak: 196.7 °C
Peak: 307.5 °C
Onset: 187.7 °C
Area: 128.4 J/g
Area: 620.9 J/g
90 min.
Peak: 197.0 °C
Peak: 310.0 °C
Onset: 188.1 °C
Area: 127.2 J/g
Area: 634.1 J/g
120 min.
Peak: 195.8 °C
Peak: 309.6 °C
Onset: 186.9 °C
Area: 127.2 J/g
Area: 620.8 J/g
Temperature /°C

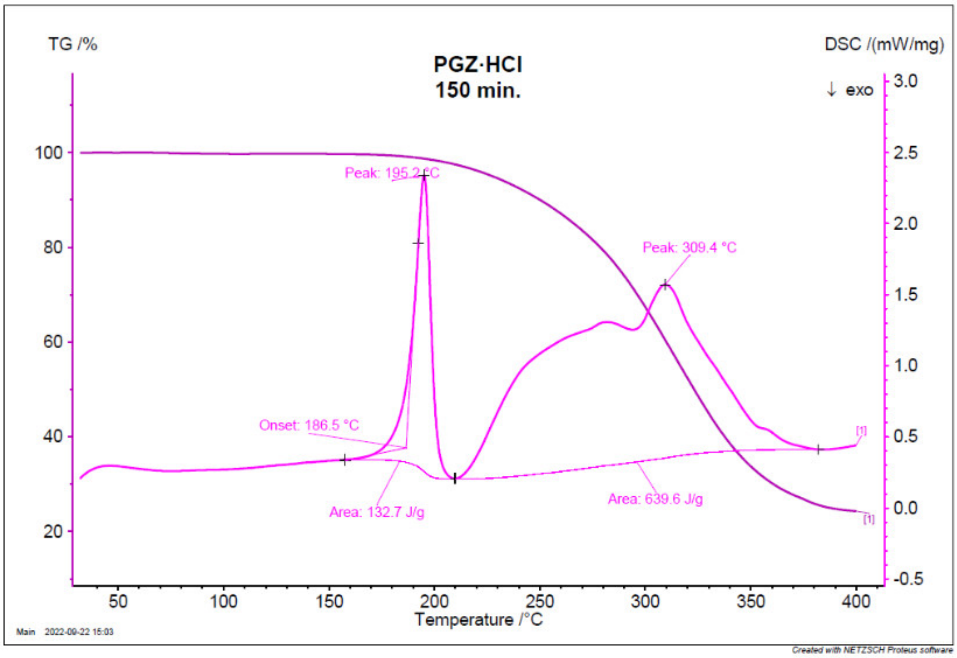

Figure S6. Evaluation of the amorphization ability of the PGZ·HCl

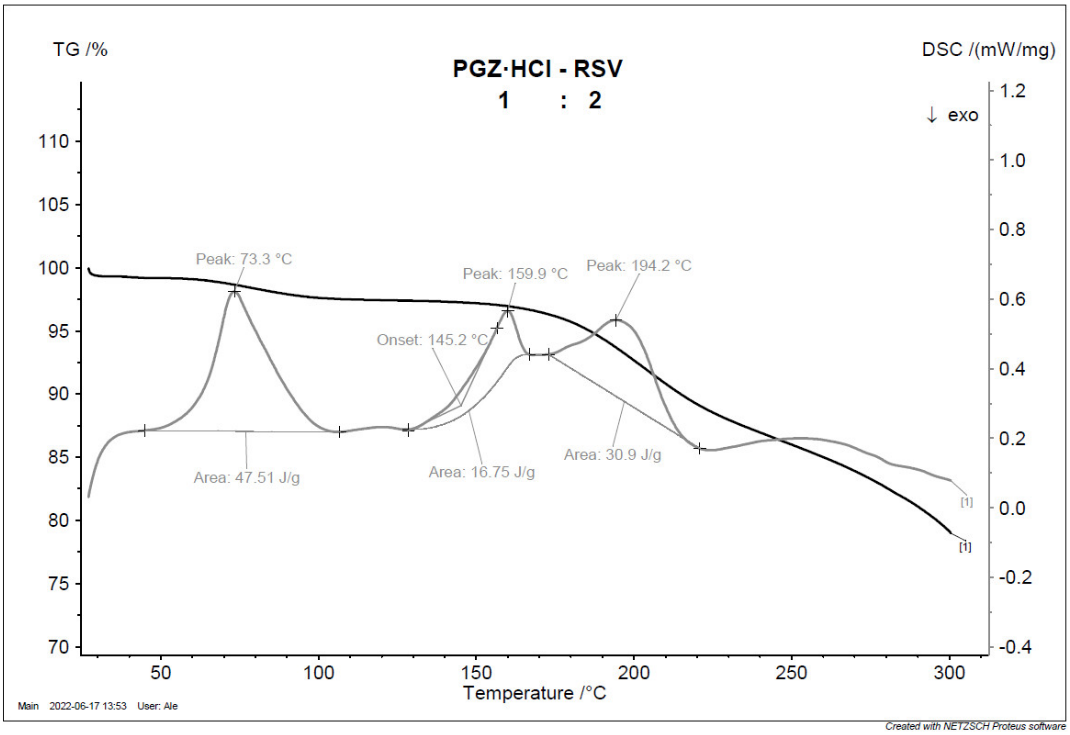
PGZ·HCl - RSV
1 : 2
TG /%
DSC /(mW/mg)
↓ exo
Peak: 73.3 °C
Peak: 159.9 °C
Peak: 194.2 °C
Onset: 145.2 °C
Area: 47.51 J/g
Area: 16.75 J/g
Area: 30.9 J/g
Temperature /°C
Main 2022-06-17 13:53 User: Ale
Created with NETZSCH Proteus software

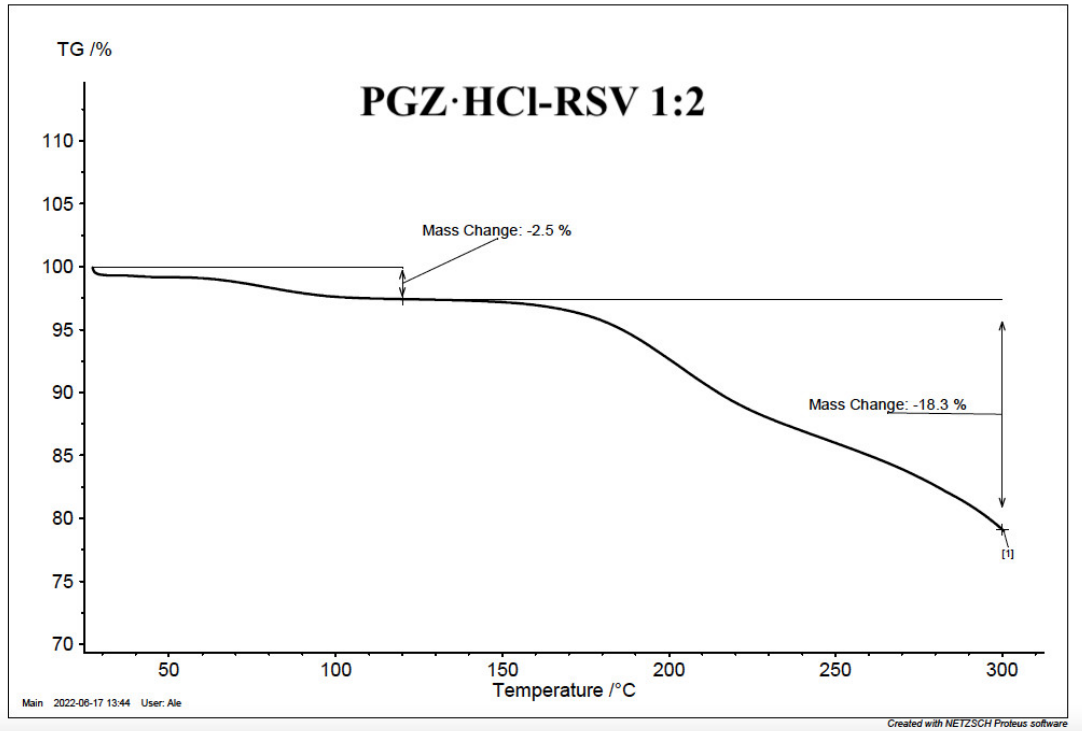
PGZ·HCl-RSV 1:2
TG /%
Mass Change: -2.5 %
Mass Change: -18.3 %
Temperature /°C
Main 2022-06-17 13:44 User: Ale
Created with NETZSCH Proteus software

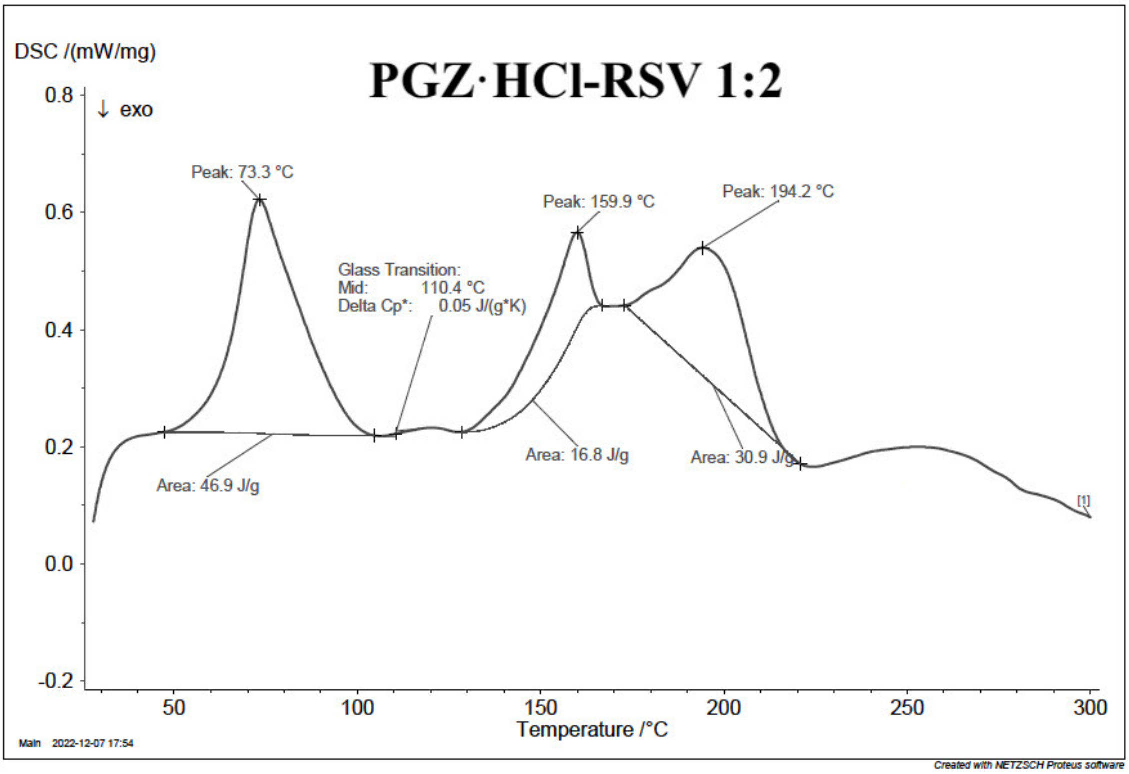

DSC /(mW/mg)
PGZ·HCl-RSV 1:2
↓ exo
Peak: 73.3 °C
Peak: 159.9 °C
Peak: 194.2 °C
Glass Transition:
Mid: 110.4 °C
Delta Cp*: 0.05 J/(g*K)
Area: 46.9 J/g
Area: 16.8 J/g
Area: 30.9 J/g
[1]
0.8
0.6
0.4
0.2
0.0
-0.2
50
100
150
200
250
300
Temperature /°C
Main 2022-12-07 17:54
Created with NETZSCH Proteus software

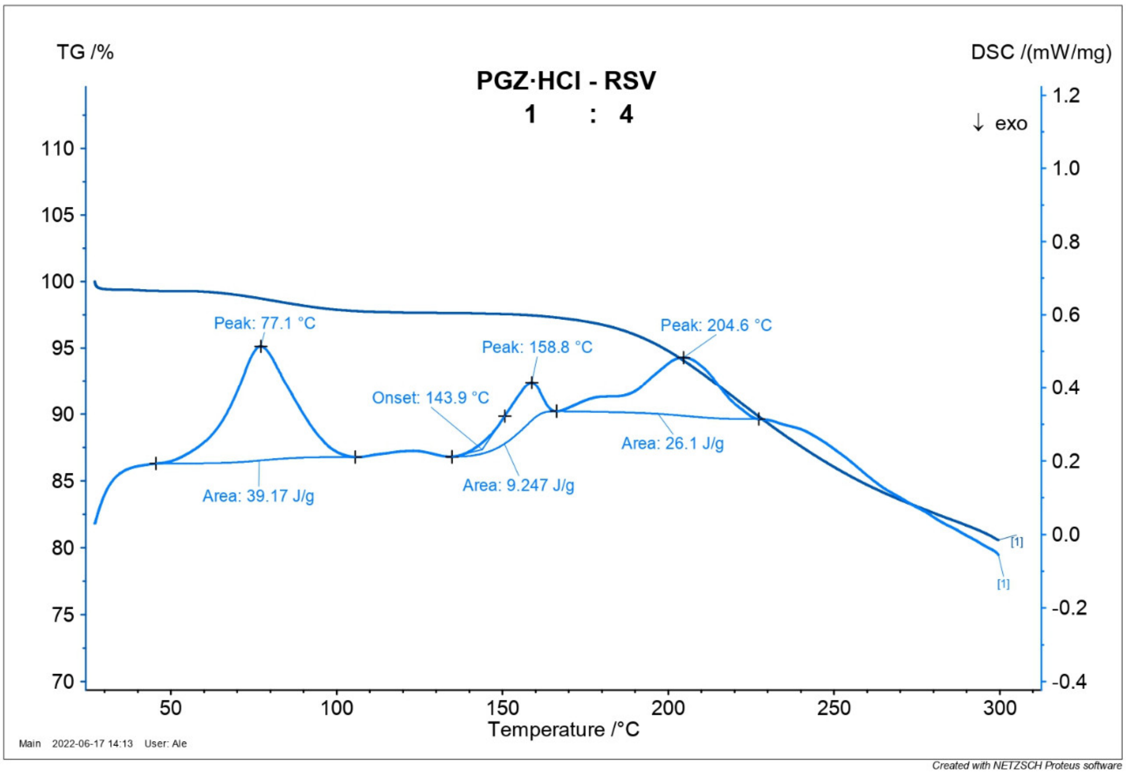

TG /%
DSC /(mW/mg)
PGZ·HCl - RSV
1 : 4
↓ exo
Peak: 77.1 °C
Peak: 158.8 °C
Peak: 204.6 °C
Onset: 143.9 °C
Area: 39.17 J/g
Area: 9.247 J/g
Area: 26.1 J/g
[1]
[1]
110
105
100
95
90
85
80
75
70
1.2
1.0
0.8
0.6
0.4
0.2
0.0
-0.2
-0.4
50
100
150
200
250
300
Temperature /°C
Main 2022-06-17 14:13 User: Ale
Created with NETZSCH Proteus software

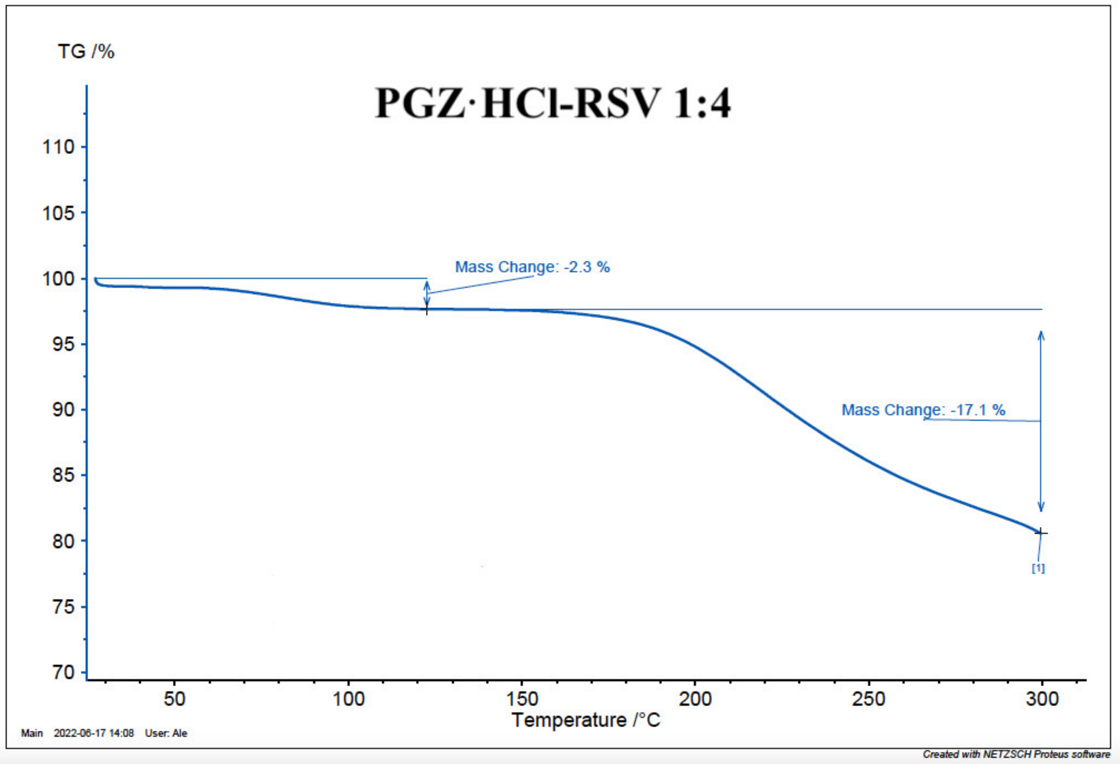

TG /%
PGZ·HCl-RSV 1:4
110
105
100
95
90
85
80
75
70
Mass Change: -2.3 %
Mass Change: -17.1 %
[1]
50
100
150
200
250
300
Temperature /°C
Main 2022-06-17 14:08 User: Ale
Created with NETZSCH Proteus software

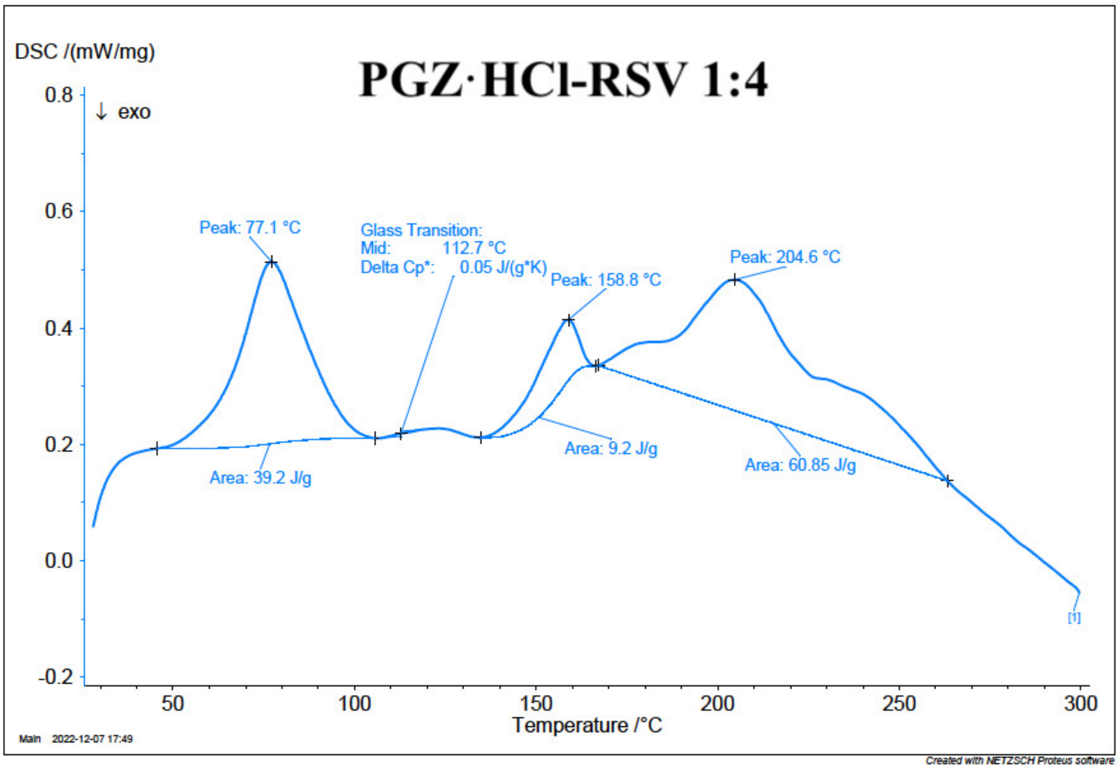

DSC /(mW/mg)
PGZ·HCl-RSV 1:4
↓ exo
0.8
0.6
0.4
0.2
0.0
-0.2
Peak: 77.1 °C
Glass Transition:
Mid: 112.7 °C
Delta Cp*: 0.05 J/(g*K)
Peak: 158.8 °C
Peak: 204.6 °C
Area: 39.2 J/g
Area: 9.2 J/g
Area: 60.85 J/g
[1]
50
100
150
200
250
300
Temperature /°C
Main 2022-12-07 17:49
Created with NETZSCH Proteus software

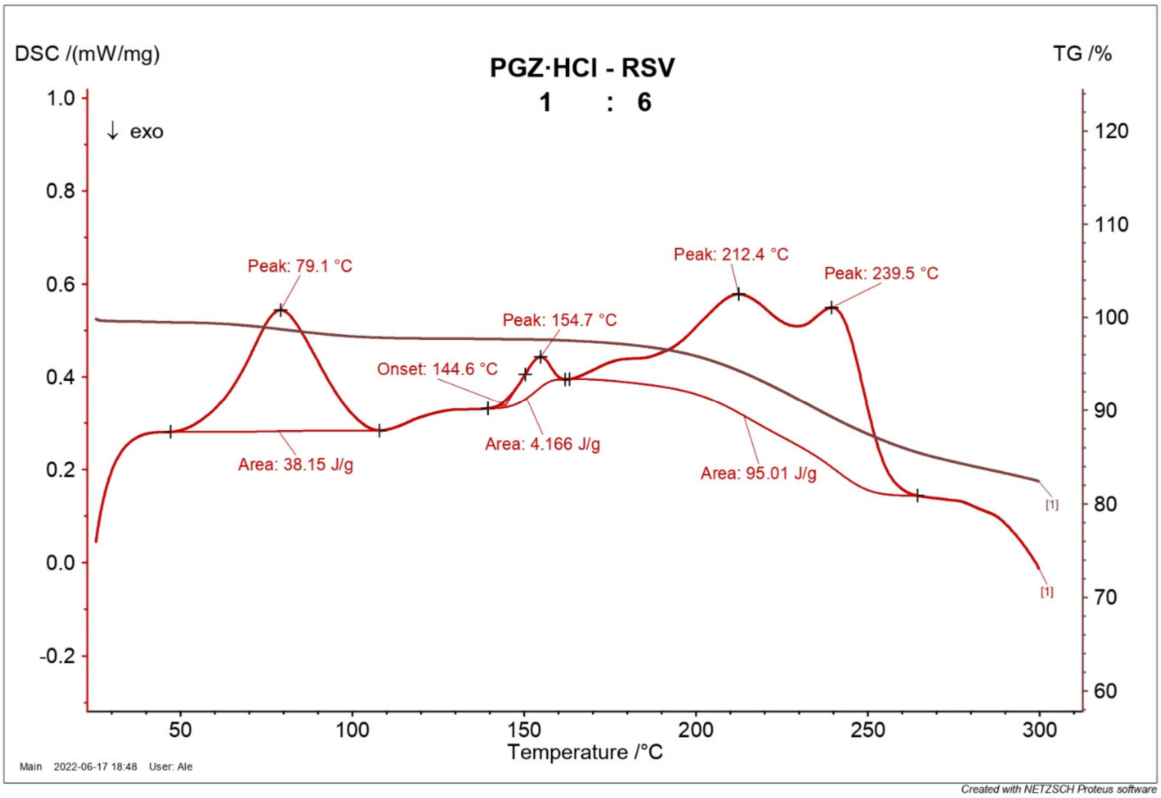
DSC /(mW/mg)
PGZ·HCl - RSV
1 : 6
TG /%
↓ exo
Peak: 79.1 °C
Peak: 212.4 °C
Peak: 239.5 °C
Peak: 154.7 °C
Onset: 144.6 °C
Area: 38.15 J/g
Area: 4.166 J/g
Area: 95.01 J/g
Temperature /°C
Main 2022-06-17 18:48 User: Ale
Created with NETZSCH Proteus software

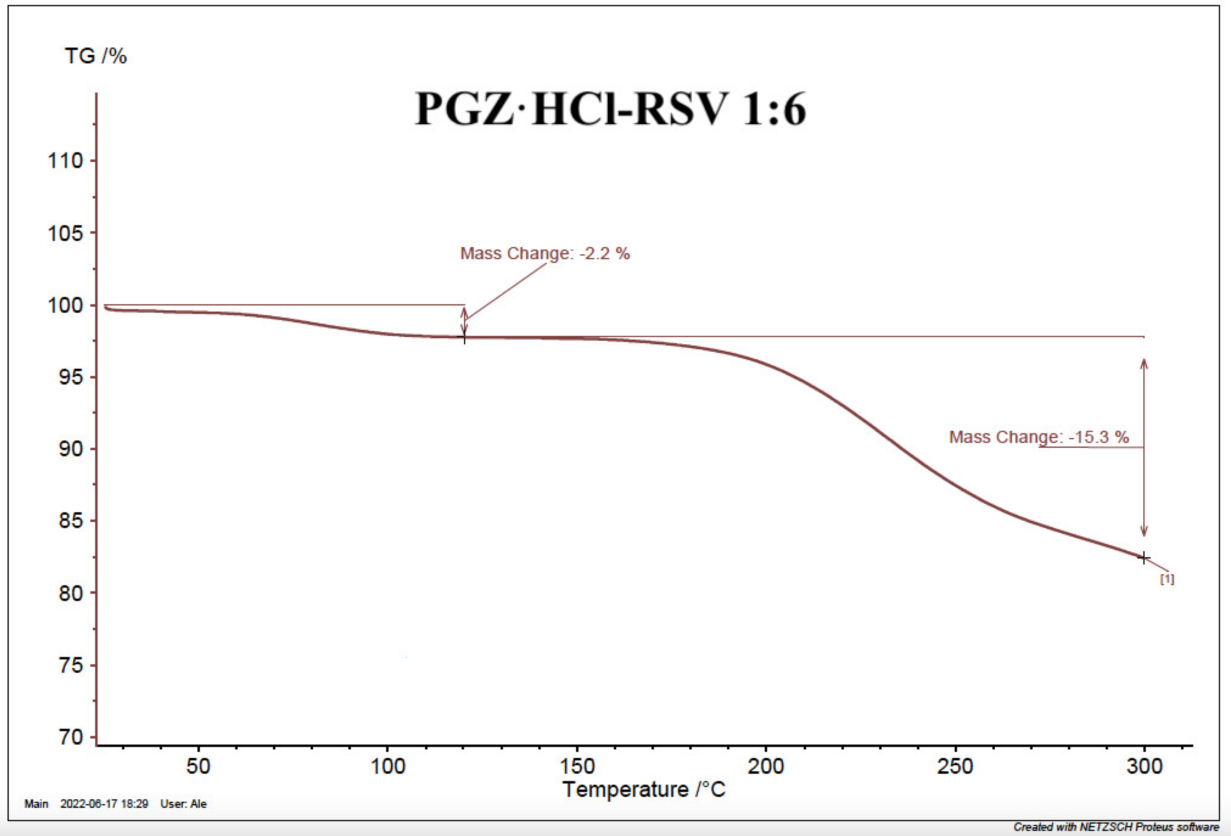
TG /%
PGZ·HCl-RSV 1:6
Mass Change: -2.2 %
Mass Change: -15.3 %
Temperature /°C
Main 2022-06-17 18:29 User: Ale
Created with NETZSCH Proteus software

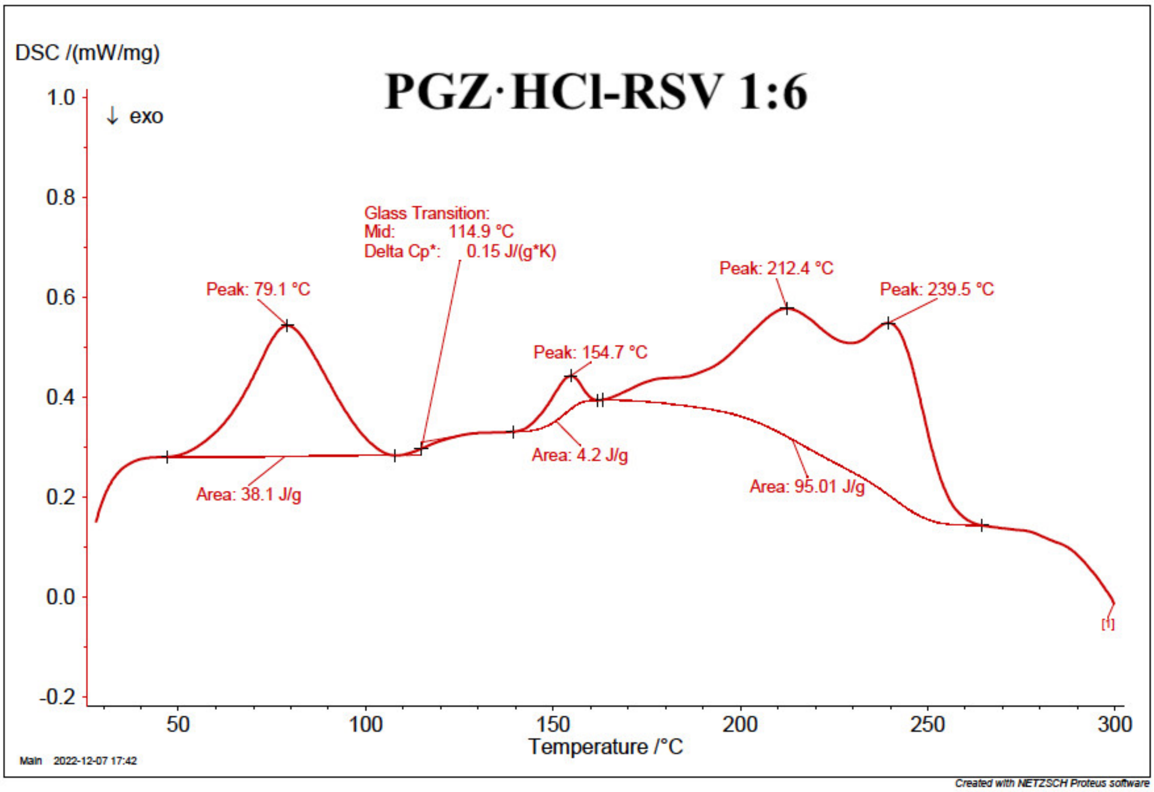
DSC /(mW/mg)
PGZ·HCl-RSV 1:6
↓ exo
Glass Transition:
Mid: 114.9 °C
Delta Cp*: 0.15 J/(g*K)
Peak: 79.1 °C
Peak: 154.7 °C
Peak: 212.4 °C
Peak: 239.5 °C
Area: 38.1 J/g
Area: 4.2 J/g
Area: 95.01 J/g
Temperature /°C
Main 2022-12-07 17:42
Created with NETZSCH Proteus software

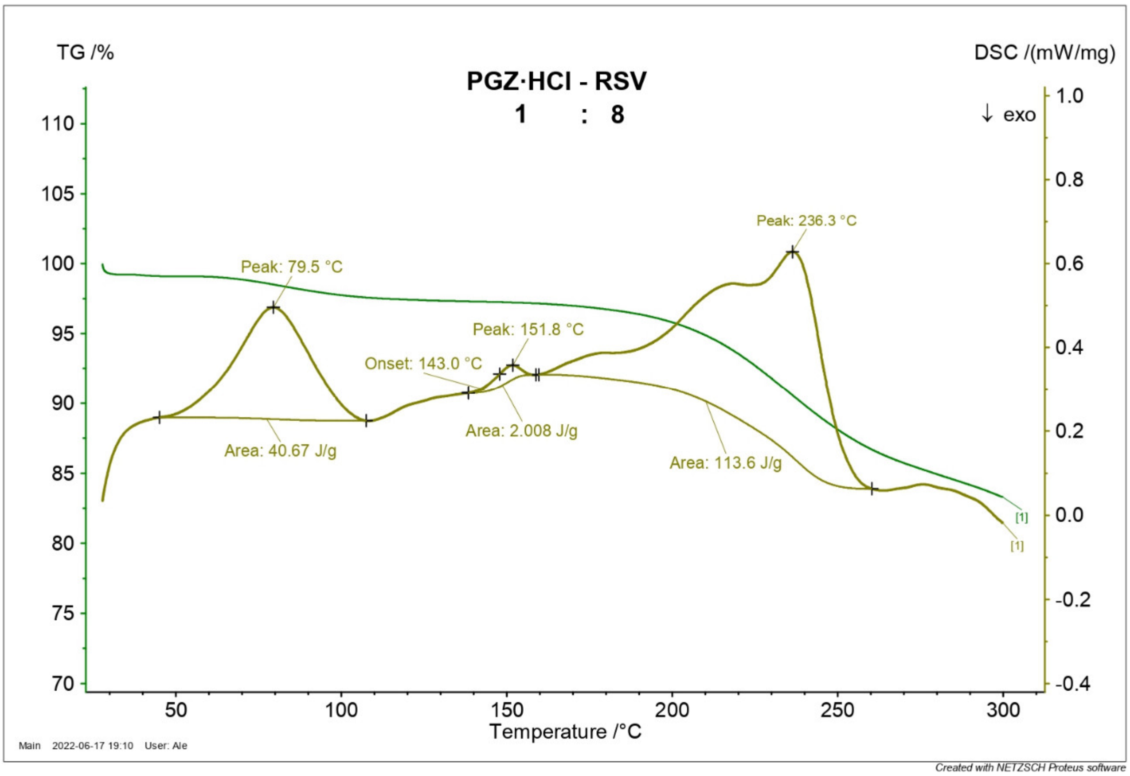
TG /%
DSC /(mW/mg)
PGZ·HCl - RSV
1 : 8
↓ exo
Peak: 79.5 °C
Onset: 143.0 °C
Peak: 151.8 °C
Peak: 236.3 °C
Area: 40.67 J/g
Area: 2.008 J/g
Area: 113.6 J/g
Temperature /°C
Main 2022-06-17 19:10 User: Ale
Created with NETZSCH Proteus software

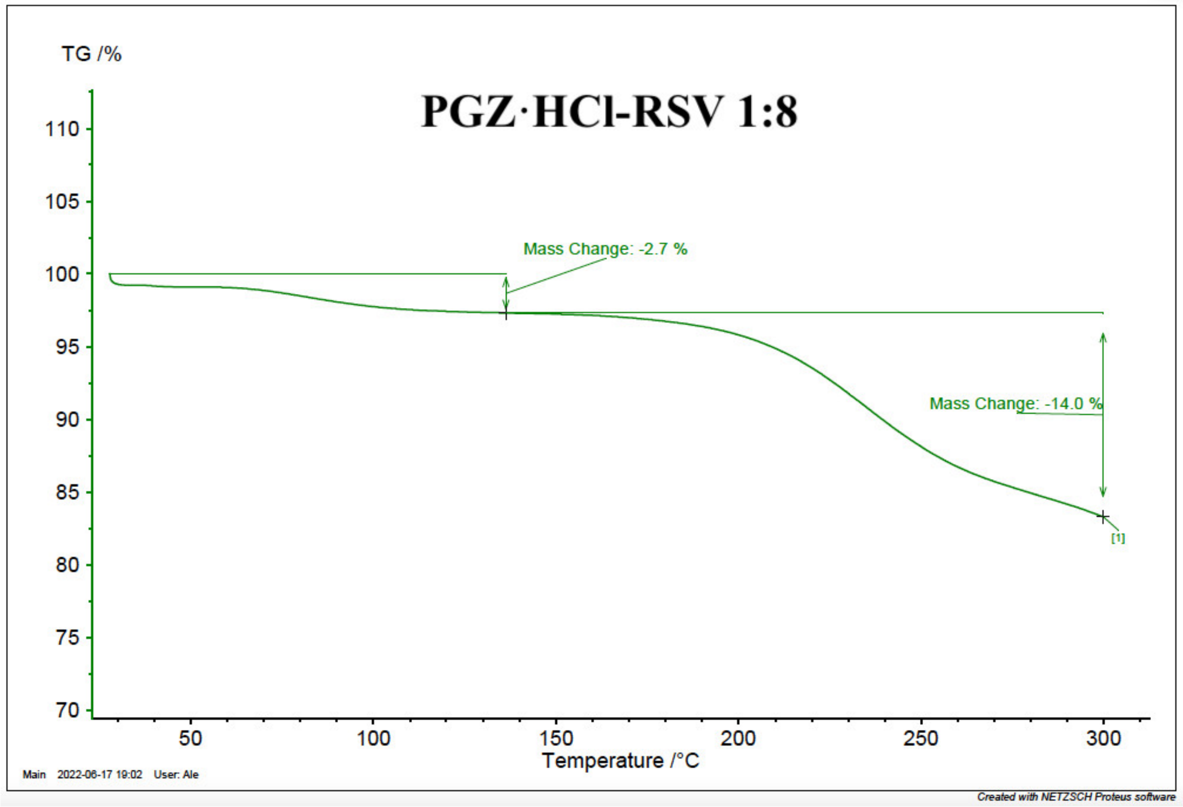
TG /%
PGZ·HCl-RSV 1:8
110
105
100
95
90
85
80
75
70
Mass Change: -2.7 %
Mass Change: -14.0 %
[1]
50
100
150
200
250
300
Temperature /°C
Main 2022-06-17 19:02 User: Ale
Created with NETZSCH Proteus software

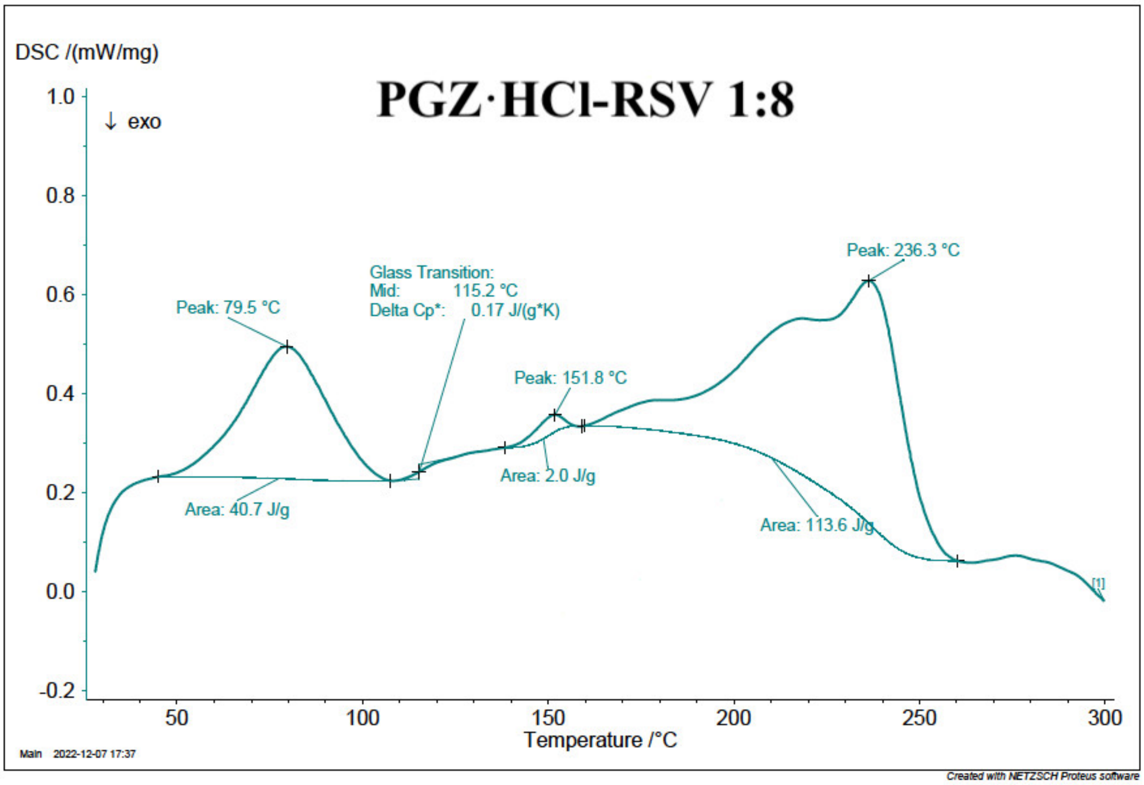
DSC /(mW/mg)
↓ exo
PGZ·HCl-RSV 1:8
1.0
0.8
0.6
0.4
0.2
0.0
-0.2
Peak: 79.5 °C
Glass Transition:
Mid: 115.2 °C
Delta Cp*: 0.17 J/(g*K)
Peak: 151.8 °C
Peak: 236.3 °C
Area: 40.7 J/g
Area: 2.0 J/g
Area: 113.6 J/g
[1]
50
100
150
200
250
300
Temperature /°C
Main 2022-12-07 17:37
Created with NETZSCH Proteus software

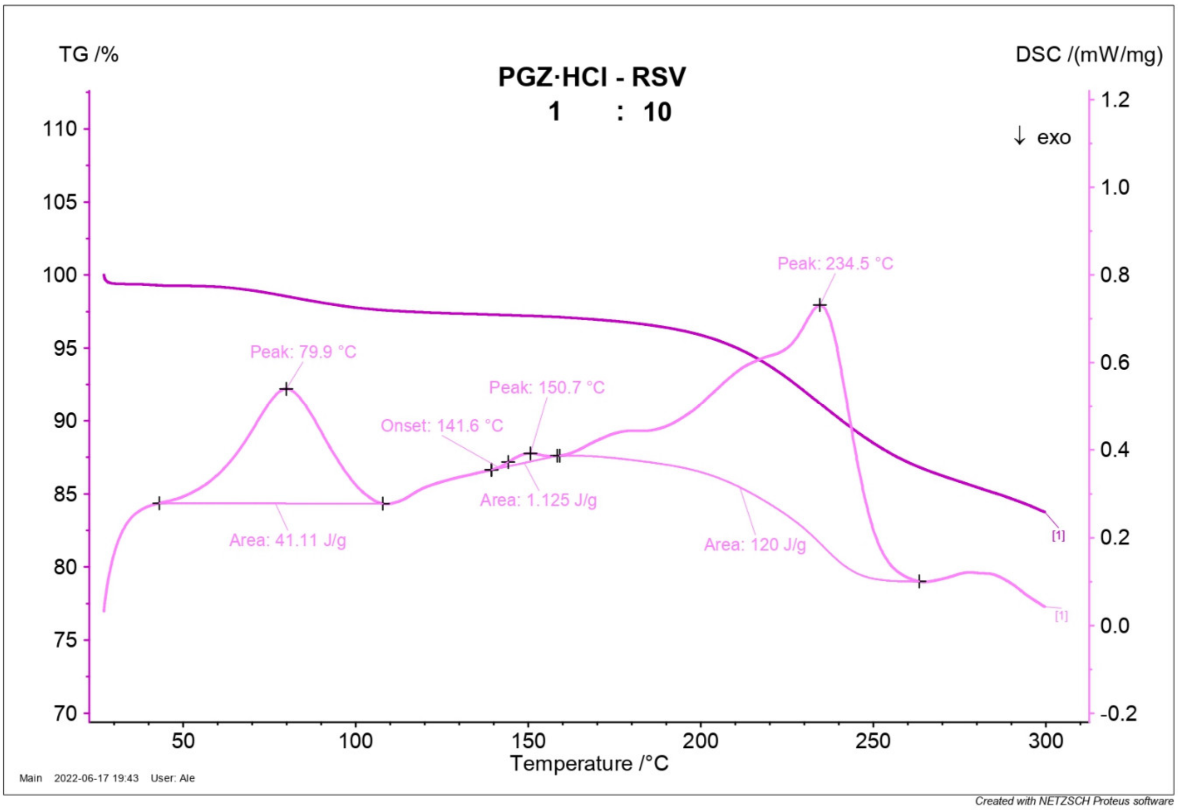
TG /%
PGZ·HCl - RSV
1 : 10
DSC /(mW/mg)
↓ exo
Peak: 234.5 °C
Peak: 79.9 °C
Peak: 150.7 °C
Onset: 141.6 °C
Area: 1.125 J/g
Area: 41.11 J/g
Area: 120 J/g
[1]
[1]
Temperature /°C
Main 2022-06-17 19:43 User: Ale
Created with NETZSCH Proteus software

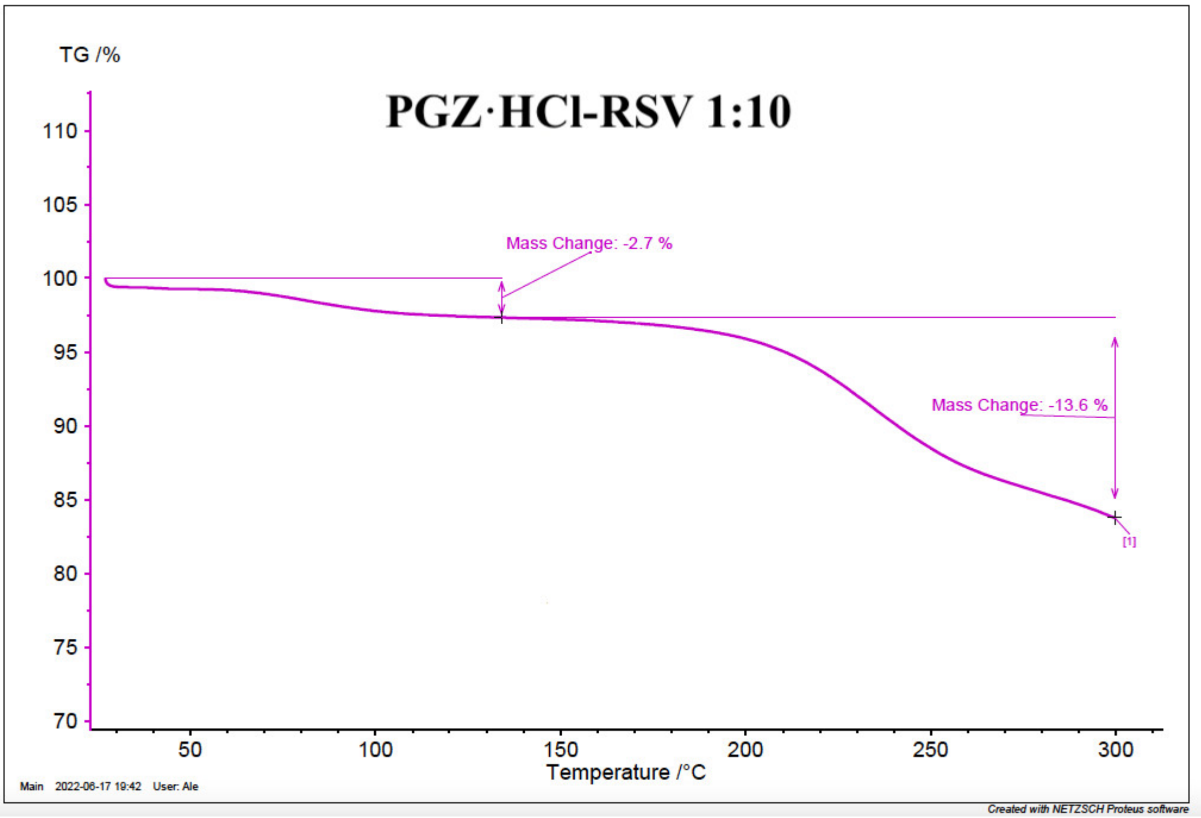
TG /%
PGZ·HCl-RSV 1:10
Mass Change: -2.7 %
Mass Change: -13.6 %
[1]
Temperature /°C
Main 2022-06-17 19:42 User: Ale
Created with NETZSCH Proteus software

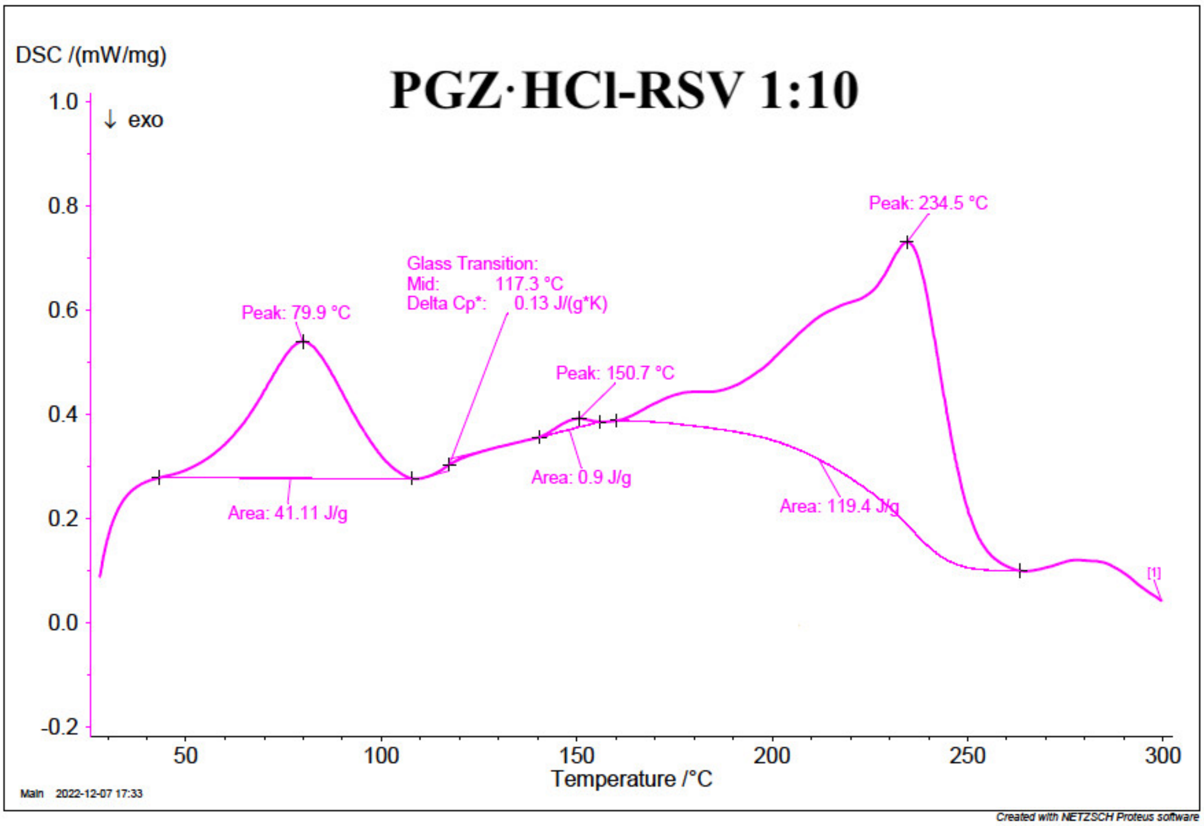

Figure S7. Individual DSC-TGA thermograms of the solid forms of PGZ·HCl-RSV at different stoichiometric ratios (1:2; 1:4; 1:6; 1:8 and 1:10

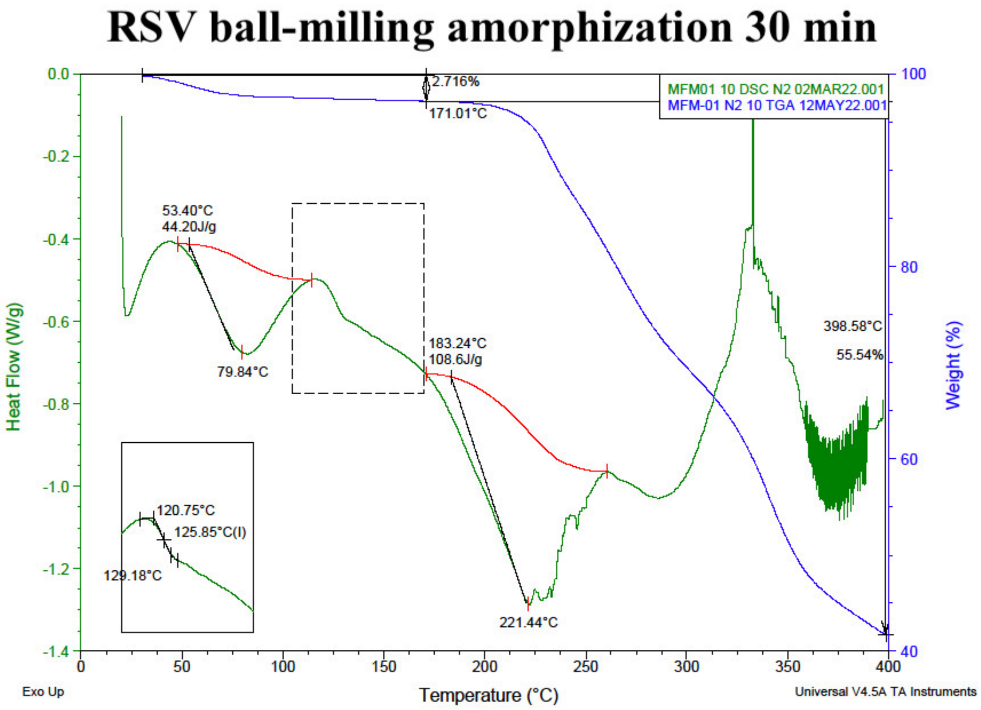
RSV ball-milling amorphization 30 min
MFM01 10 DSC N2 02MAR22.001
MFM-01 N2 10 TGA 12MAY22.001
2.716%
171.01°C
53.40°C
44.20J/g
79.84°C
183.24°C
108.6J/g
221.44°C
398.58°C
55.54%
120.75°C
125.85°C(I)
129.18°C
Heat Flow (W/g)
Weight (%)
Temperature (°C)
Exo Up
Universal V4.5A TA Instruments

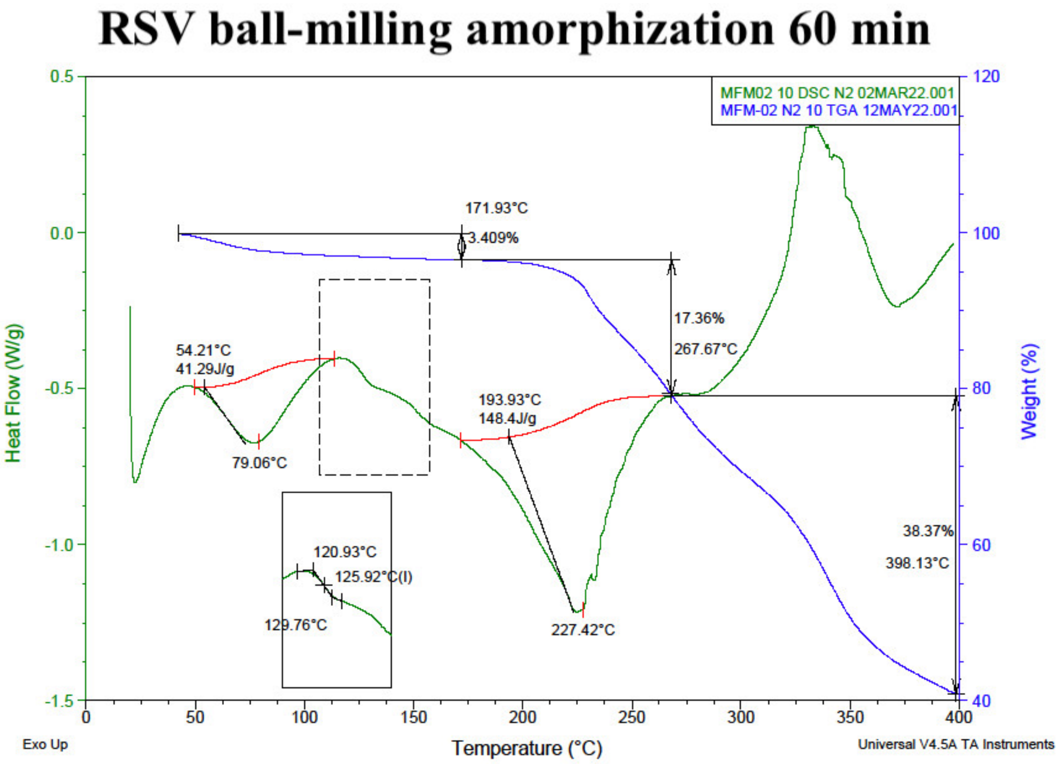
RSV ball-milling amorphization 60 min
MFM02 10 DSC N2 02MAR22.001
MFM-02 N2 10 TGA 12MAY22.001
171.93°C
3.409%
17.36%
267.67°C
54.21°C
41.29J/g
79.06°C
193.93°C
148.4J/g
227.42°C
38.37%
398.13°C
120.93°C
125.92°C(I)
129.76°C
Heat Flow (W/g)
Weight (%)
Temperature (°C)
Exo Up
Universal V4.5A TA Instruments

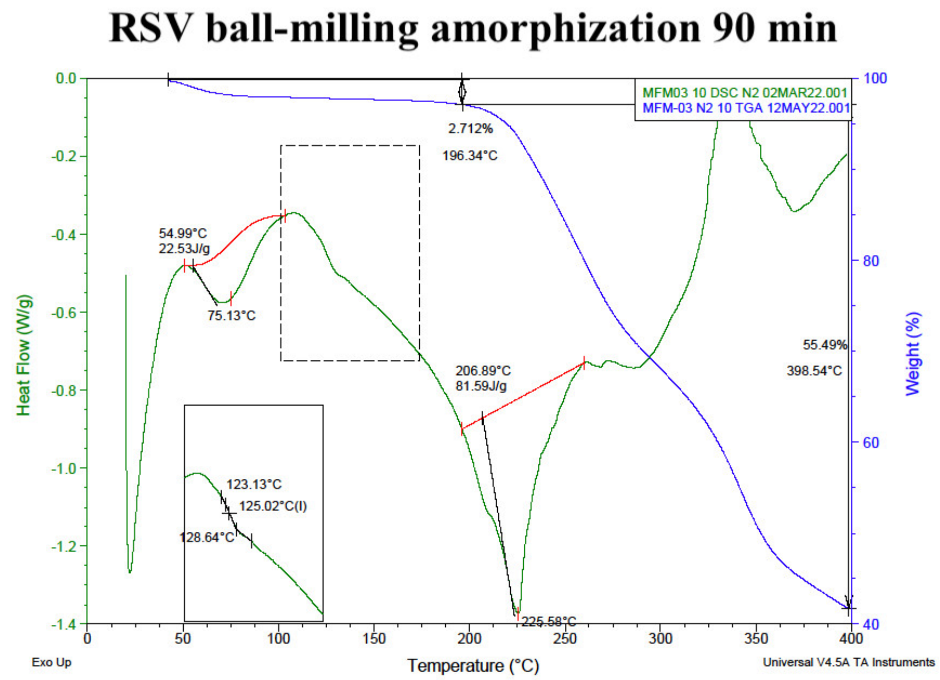
RSV ball-milling amorphization 90 min
MFM03 10 DSC N2 02MAR22.001
MFM-03 N2 10 TGA 12MAY22.001
2.712%
196.34°C
54.99°C
22.53J/g
75.13°C
206.89°C
81.59J/g
225.58°C
55.49%
398.54°C
123.13°C
125.02°C(I)
128.64°C
Heat Flow (W/g)
Weight (%)
Temperature (°C)
Exo Up
Universal V4.5A TA Instruments

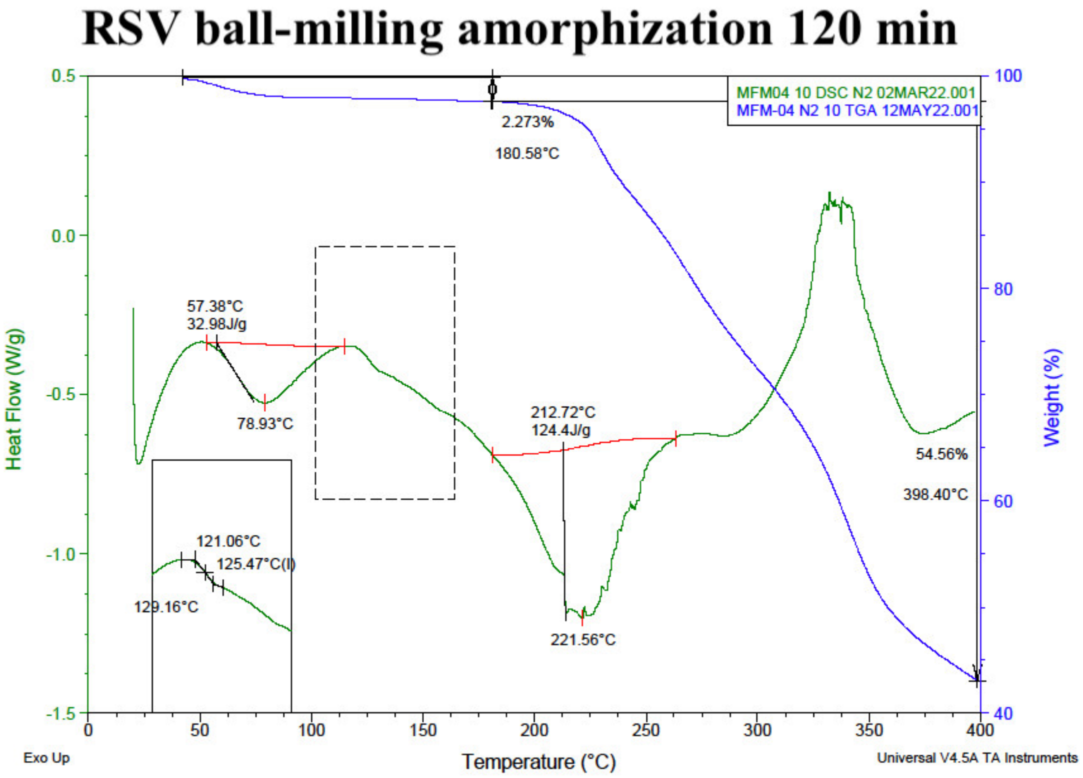
RSV ball-milling amorphization 120 min
MFM04 10 DSC N2 02MAR22.001
MFM-04 N2 10 TGA 12MAY22.001
2.273%
180.58°C
57.38°C
32.98J/g
78.93°C
212.72°C
124.4J/g
221.56°C
54.56%
398.40°C
121.06°C
125.47°C(I)
129.16°C
Heat Flow (W/g)
Weight (%)
Temperature (°C)
Exo Up
Universal V4.5A TA Instruments

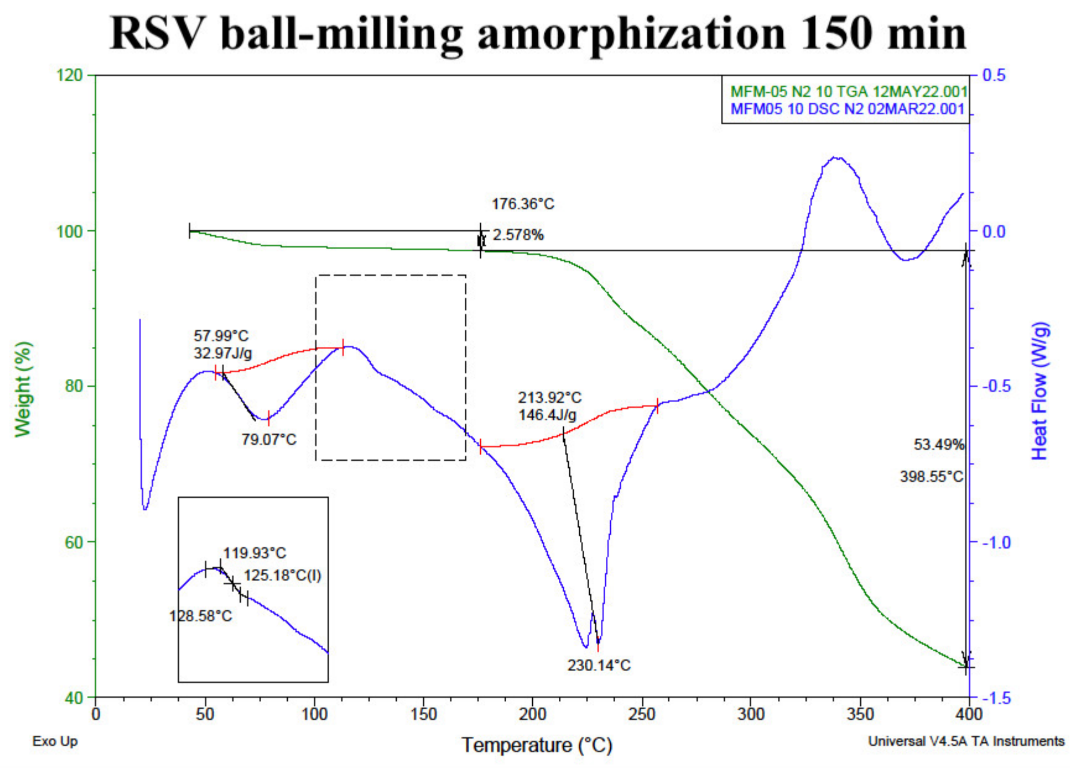

Figure S8. RSV amorphization at different times of ball-milling.

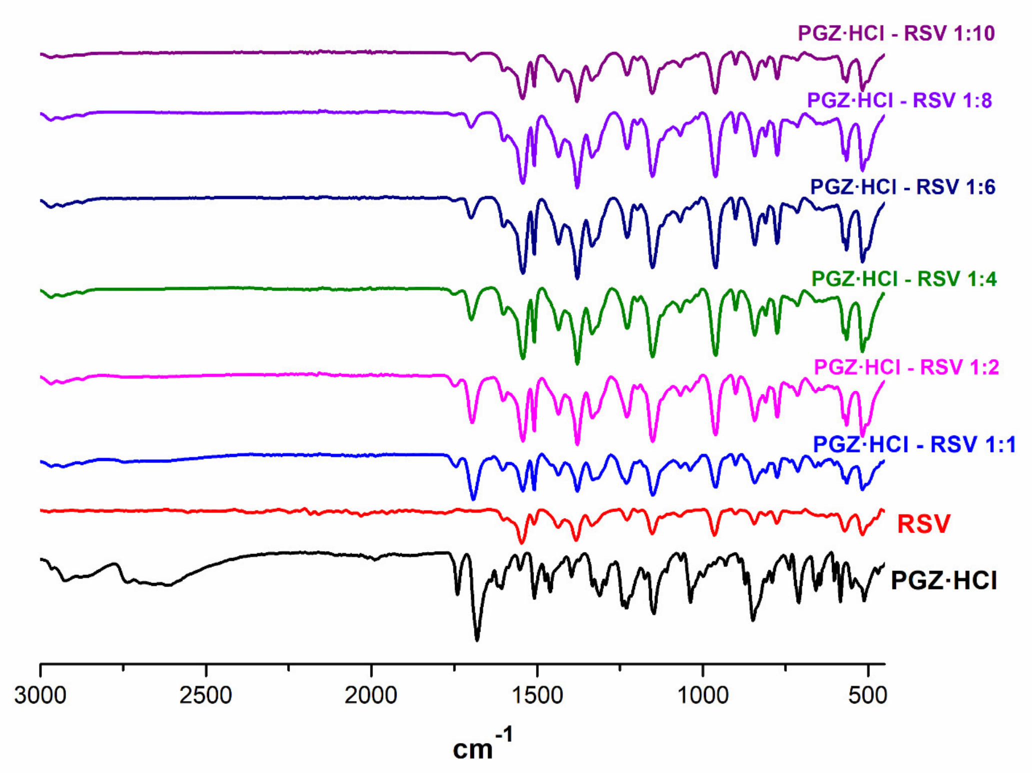

Figure S9. Full FT-IR spectra of the solid forms of PGZ·HCl-RSV at different stoichiometric ratios (1:1; 1:2; 1:4; 1:6; 1:8 and 1:10)

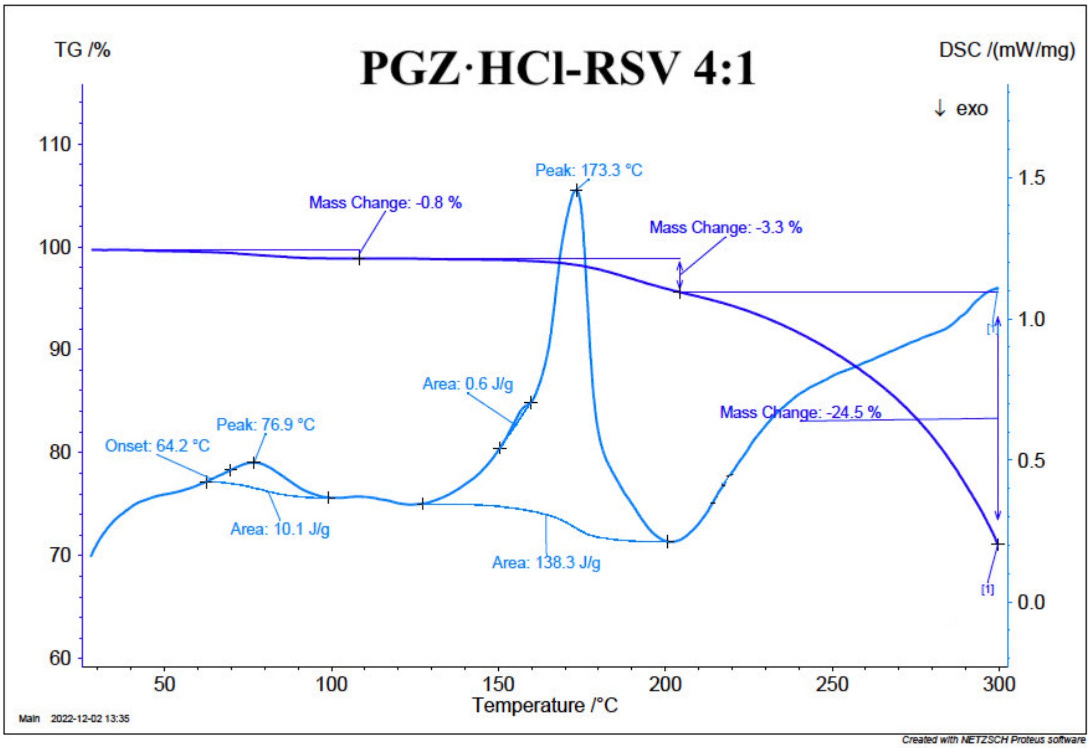

PGZ·HCl-RSV 4:1
TG /%
DSC /(mW/mg)
↓ exo
Peak: 173.3 °C
Mass Change: -0.8 %
Mass Change: -3.3 %
Area: 0.6 J/g
Peak: 76.9 °C
Onset: 64.2 °C
Mass Change: -24.5 %
Area: 10.1 J/g
Area: 138.3 J/g
[1]
Temperature /°C
Main 2022-12-02 13:35
Created with NETZSCH Proteus software

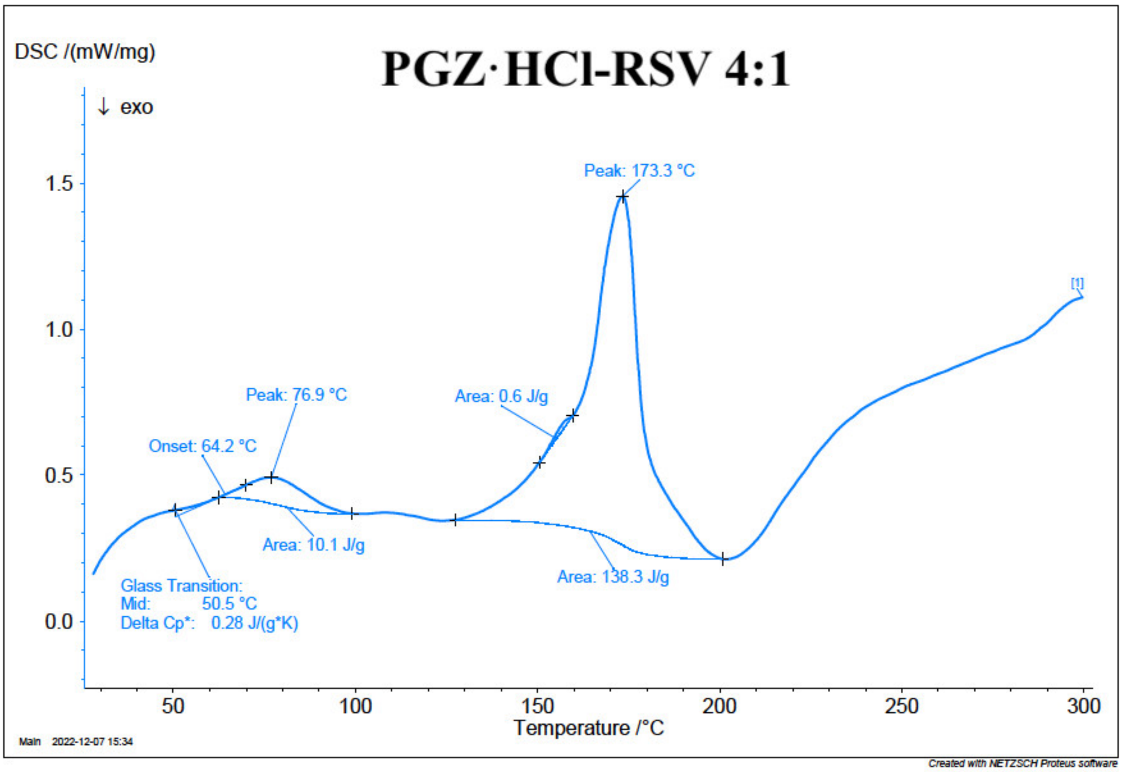

PGZ·HCl-RSV 4:1
DSC /(mW/mg)
↓ exo
Peak: 173.3 °C
[1]
Peak: 76.9 °C
Area: 0.6 J/g
Onset: 64.2 °C
Area: 10.1 J/g
Area: 138.3 J/g
Glass Transition:
Mid: 50.5 °C
Delta Cp*: 0.28 J/(g*K)
Temperature /°C
Main 2022-12-07 15:34
Created with NETZSCH Proteus software

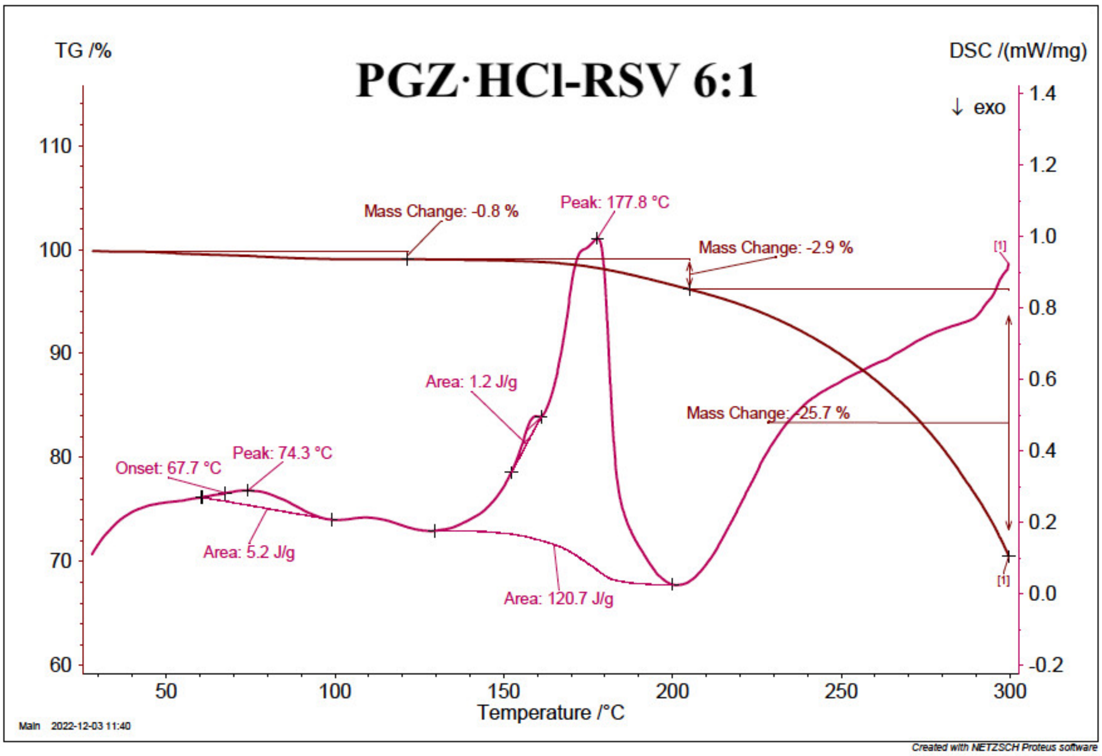
TG /%
PGZ·HCl-RSV 6:1
DSC /(mW/mg)
↓ exo
Mass Change: -0.8 %
Peak: 177.8 °C
Mass Change: -2.9 %
Area: 1.2 J/g
Mass Change: -25.7 %
Onset: 67.7 °C
Peak: 74.3 °C
Area: 5.2 J/g
Area: 120.7 J/g
[1]
Temperature /°C
Main 2022-12-03 11:40
Created with NETZSCH Proteus software

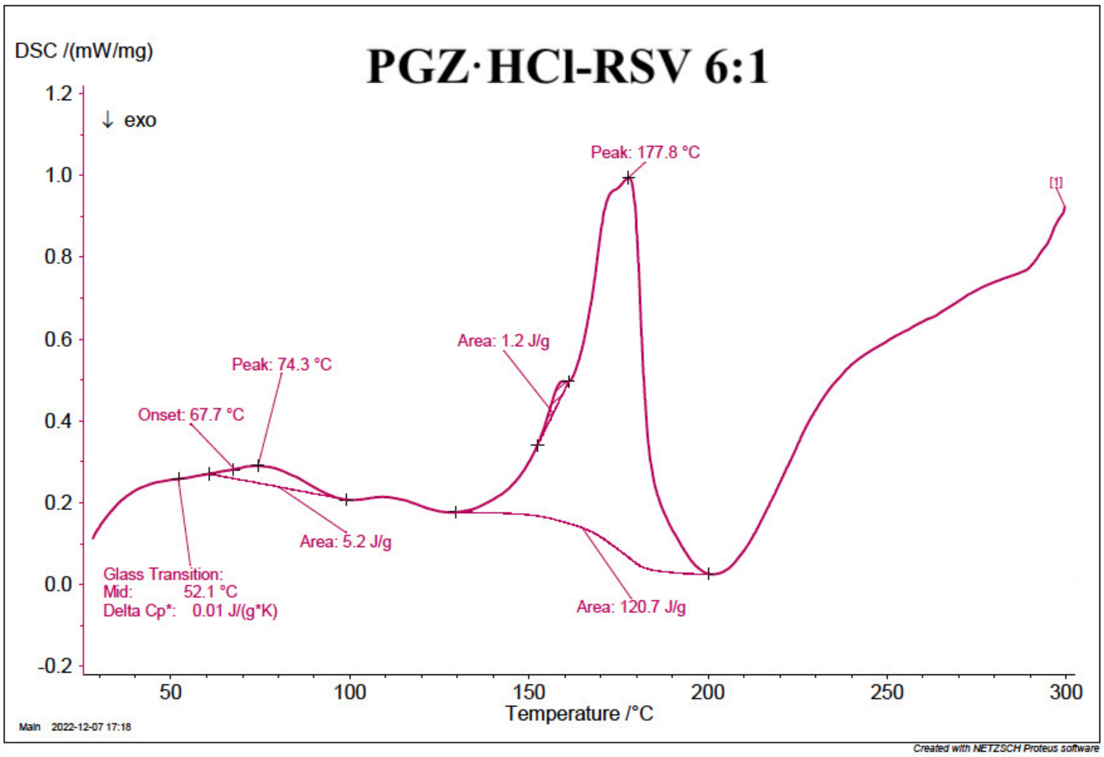
DSC /(mW/mg)
PGZ·HCl-RSV 6:1
↓ exo
Peak: 177.8 °C
[1]
Area: 1.2 J/g
Peak: 74.3 °C
Onset: 67.7 °C
Area: 5.2 J/g
Glass Transition:
Mid: 52.1 °C
Delta Cp*: 0.01 J/(g*K)
Area: 120.7 J/g
Temperature /°C
Main 2022-12-07 17:18
Created with NETZSCH Proteus software

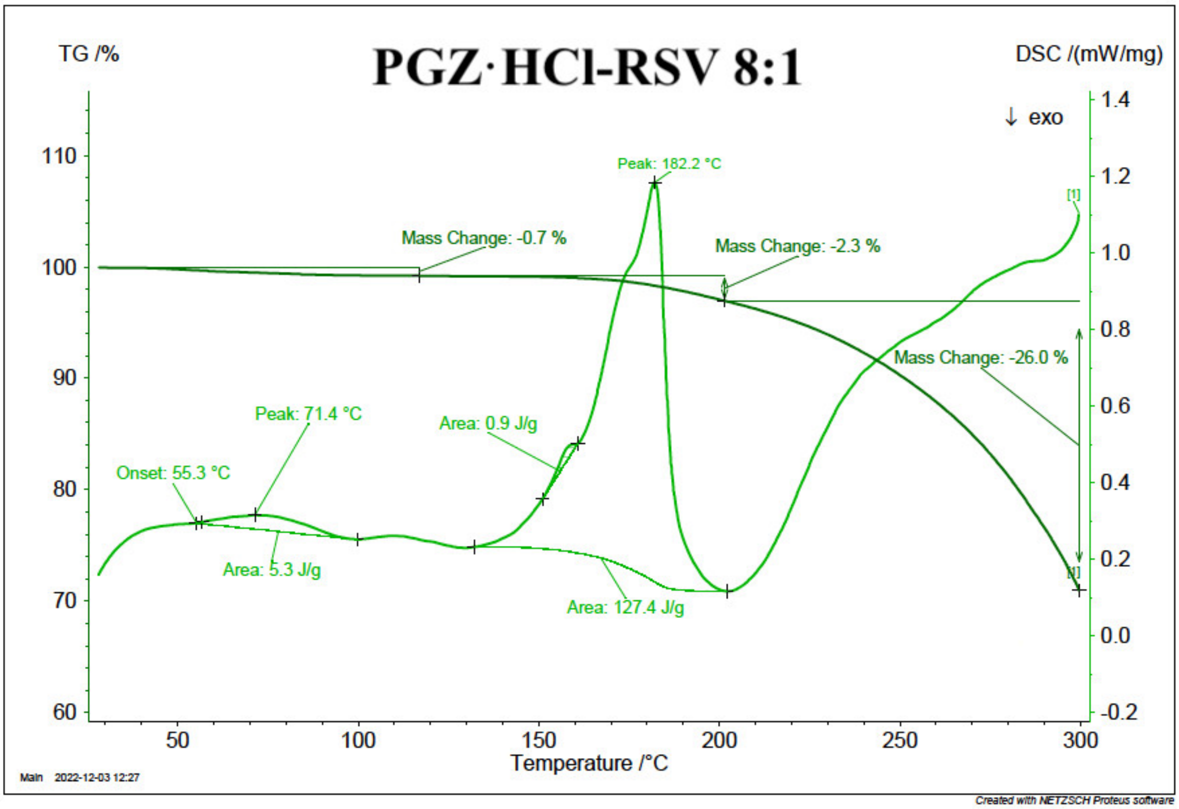

PGZ·HCl-RSV 8:1
TG /%
DSC /(mW/mg)
↓ exo
Peak: 182.2 °C
Mass Change: -0.7 %
Mass Change: -2.3 %
Mass Change: -26.0 %
Peak: 71.4 °C
Onset: 55.3 °C
Area: 0.9 J/g
Area: 5.3 J/g
Area: 127.4 J/g
[1]
Temperature /°C
Main 2022-12-03 12:27
Created with NETZSCH Proteus software

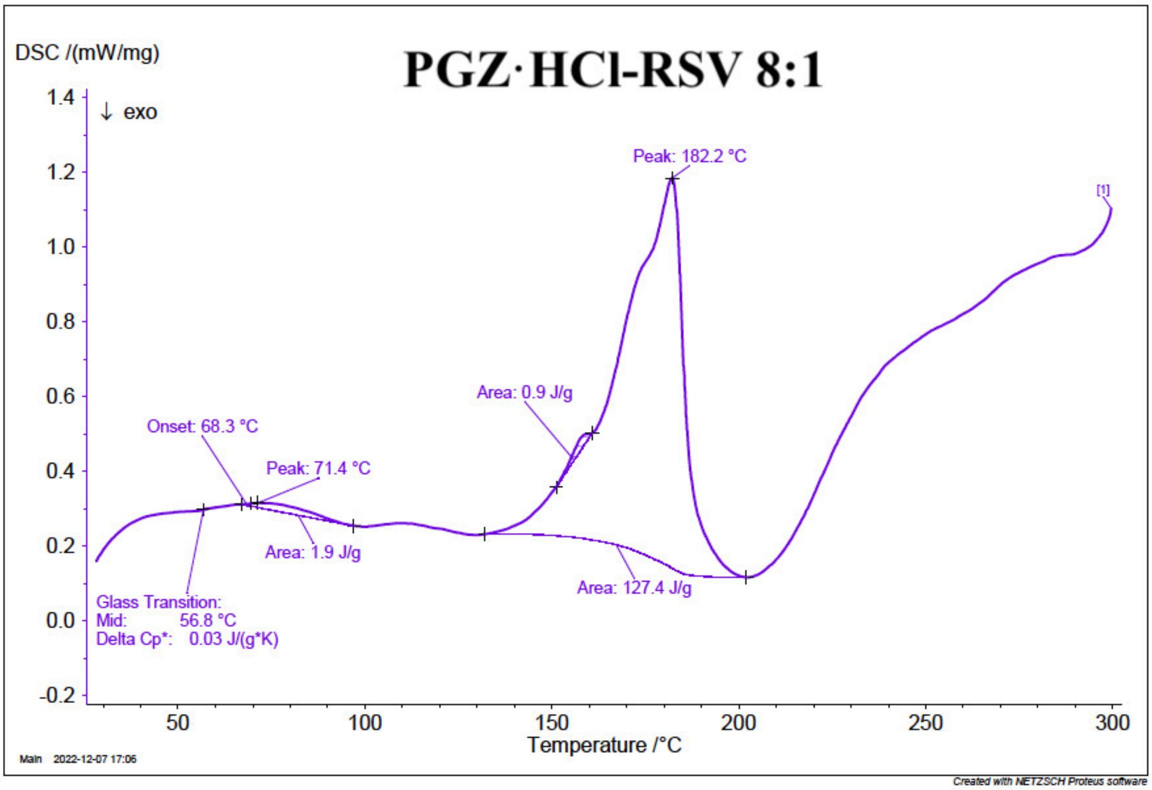

PGZ·HCl-RSV 8:1
DSC /(mW/mg)
↓ exo
Peak: 182.2 °C
Area: 0.9 J/g
Onset: 68.3 °C
Peak: 71.4 °C
Area: 1.9 J/g
Area: 127.4 J/g
Glass Transition:
Mid: 56.8 °C
Delta Cp*: 0.03 J/(g*K)
[1]
Temperature /°C
Main 2022-12-07 17:06
Created with NETZSCH Proteus software

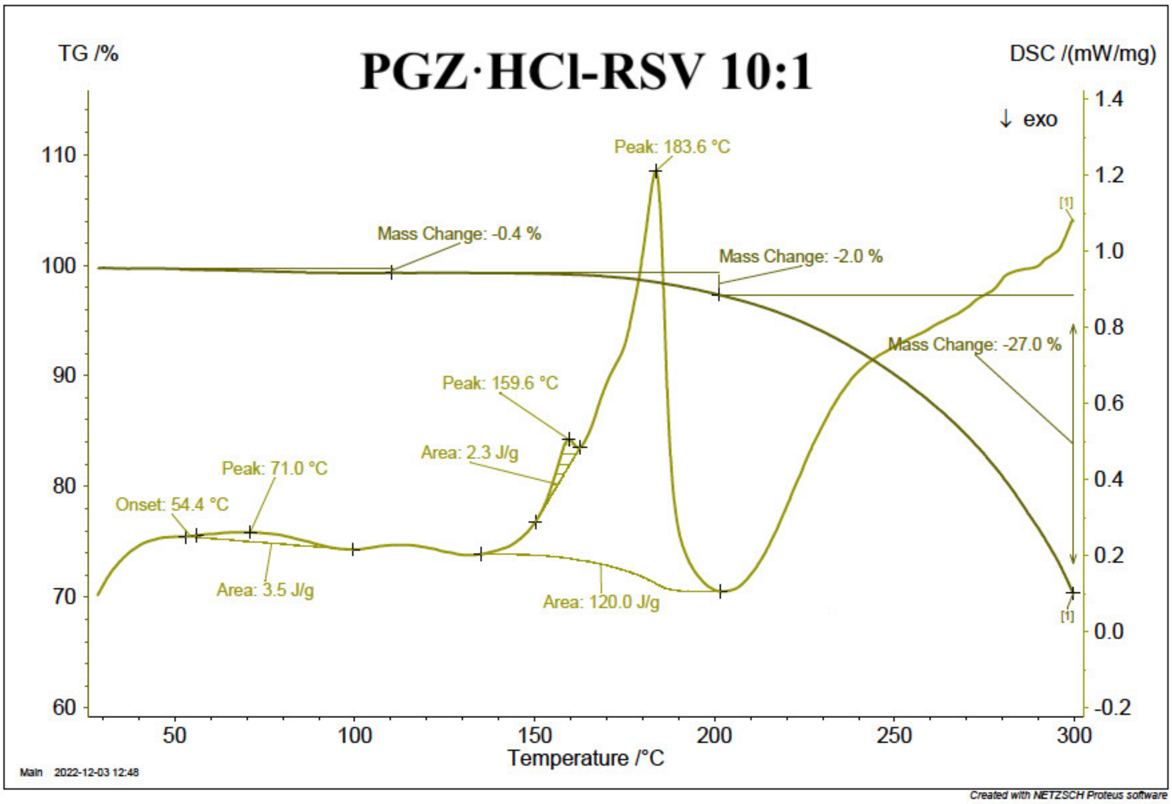

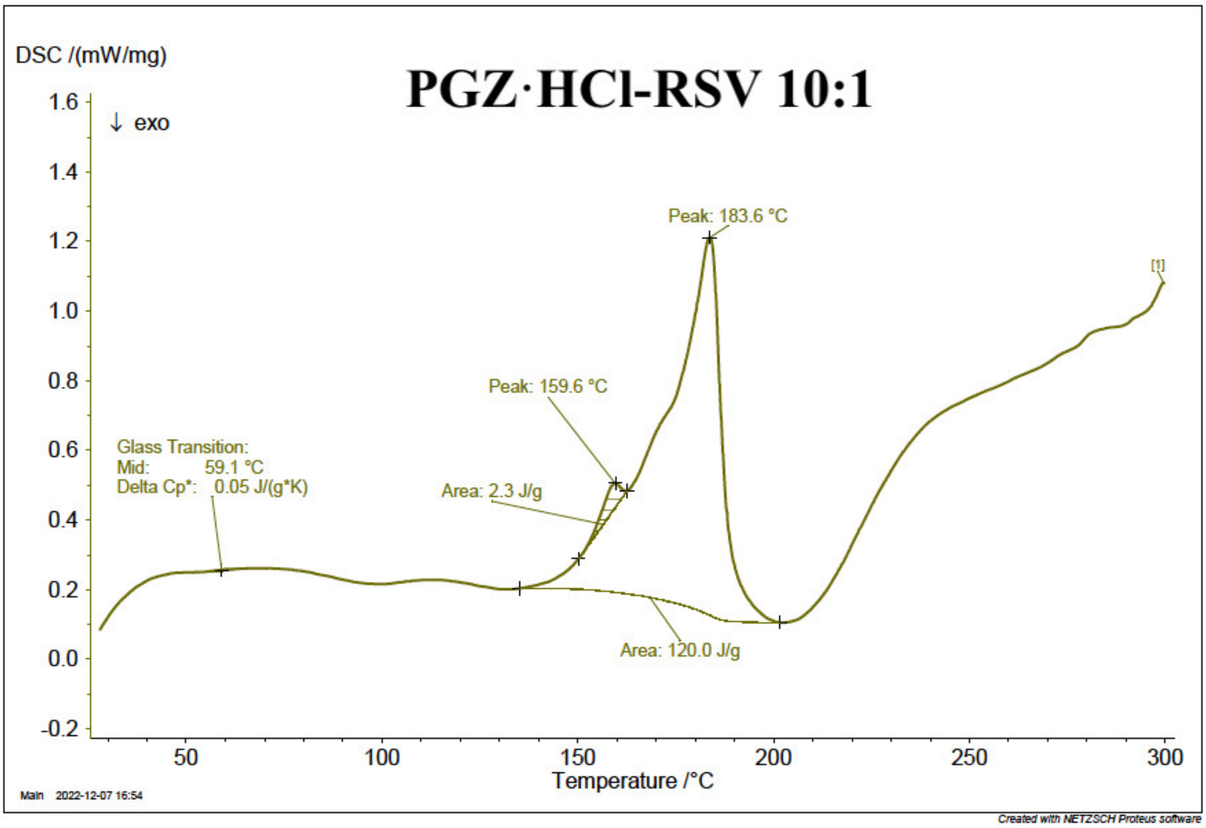

Figure S10. Individual DSC-TGA thermograms of the solid forms of PGZ·HCl-RSV at different stoichiometric ratios (4:1; 6:1; 8:1 and 10:1)

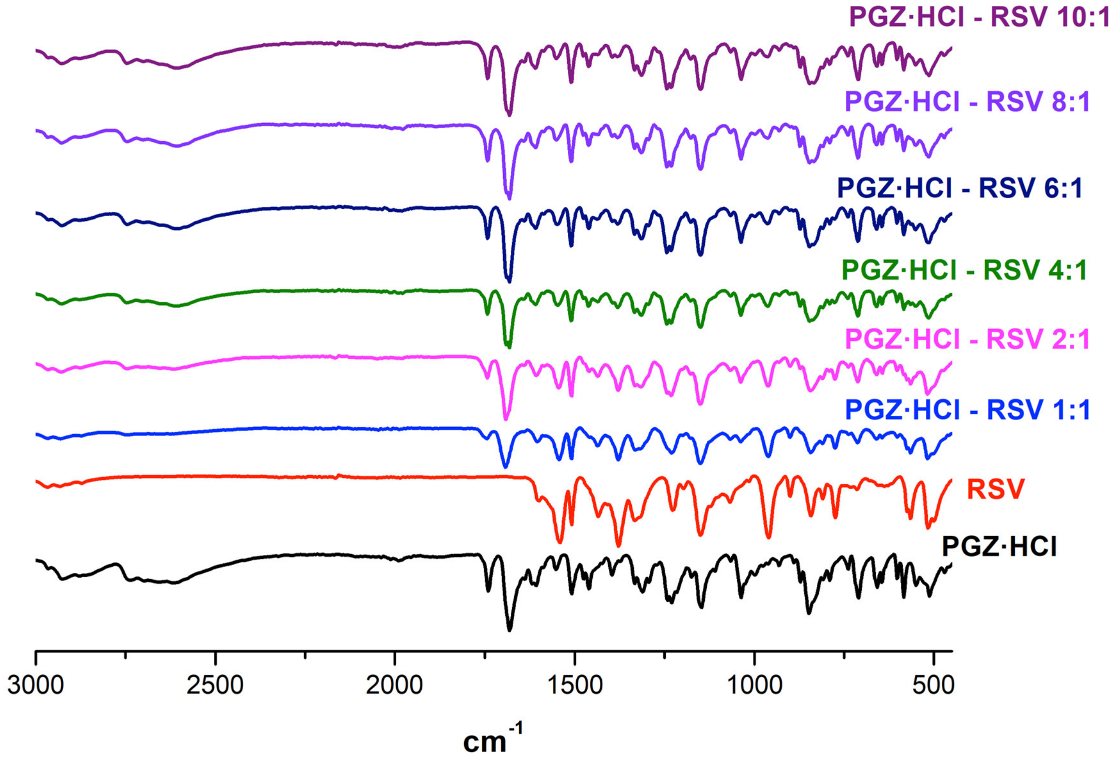

Figure S11. Full FT-IR spectra of the solid forms of PGZ·HCl-RSV at different stoichiometric ratios (1:1; 2:1; 4:1; 6:1; 8:1 and 10:1)

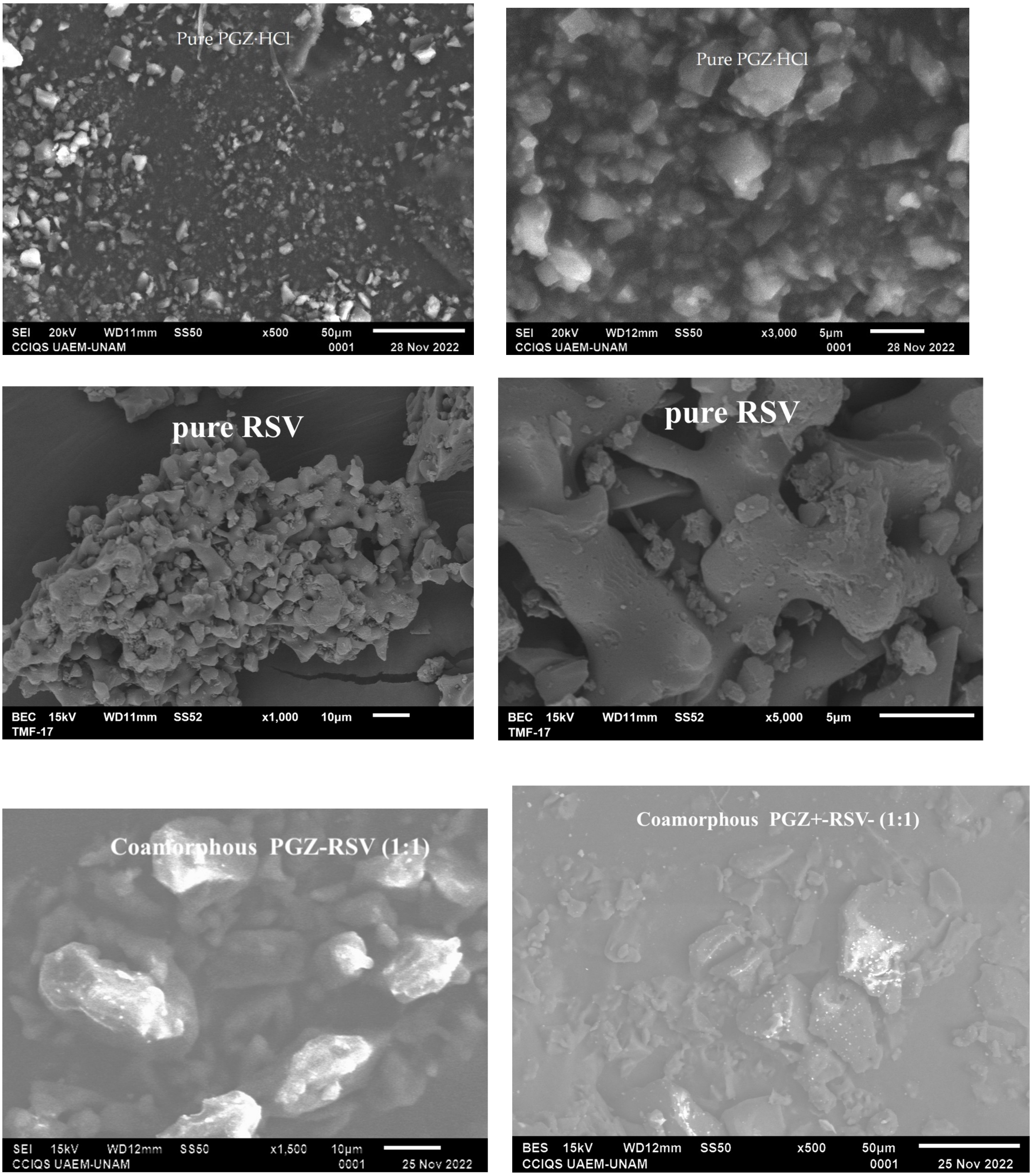
Pure PGZ·HCl
SEI 20kV WD11mm SS50 x500 50μm
CCIQS UAEM-UNAM 0001 28 Nov 2022
Pure PGZ·HCl
SEI 20kV WD12mm SS50 x3,000 5μm
CCIQS UAEM-UNAM 0001 28 Nov 2022
pure RSV
BEC 15kV WD11mm SS52 x1,000 10μm
TMF-17
pure RSV
BEC 15kV WD11mm SS52 x5,000 5μm
TMF-17
Coamorphous PGZ-RSV (1:1)
SEI 15kV WD12mm SS50 x1,500 10μm
CCIQS UAEM-UNAM 0001 25 Nov 2022
Coamorphous PGZ+-RSV- (1:1)
BES 15kV WD12mm SS50 x500 50μm
CCIQS UAEM-UNAM 0001 25 Nov 2022

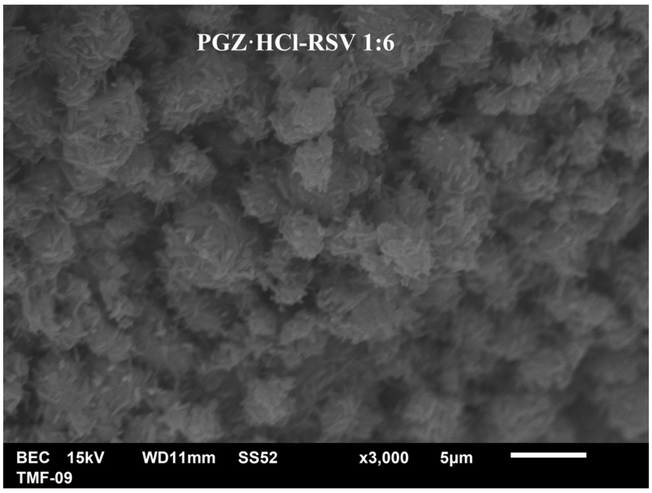
PGZ·HCl-RSV 1:6
BEC 15kV WD11mm SS52 x3,000 5µm
TMF-09

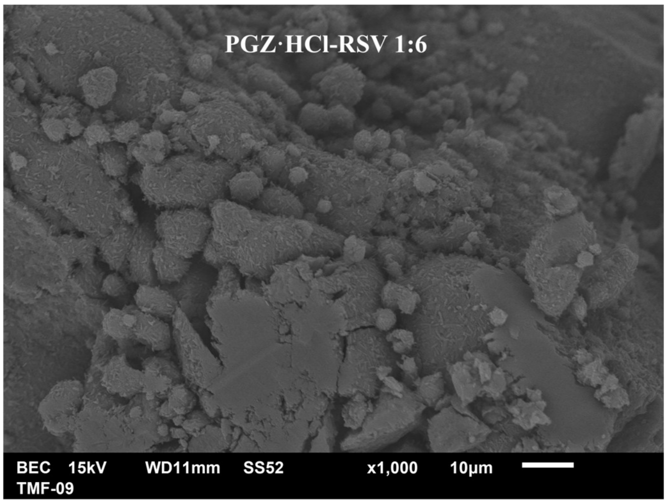
PGZ·HCl-RSV 1:6
BEC 15kV WD11mm SS52 x1,000 10µm
TMF-09

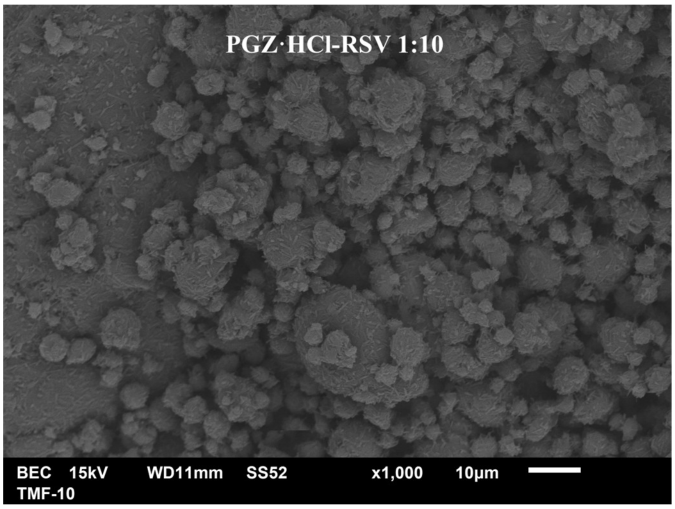
PGZ·HCl-RSV 1:10
BEC 15kV WD11mm SS52 x1,000 10µm
TMF-10

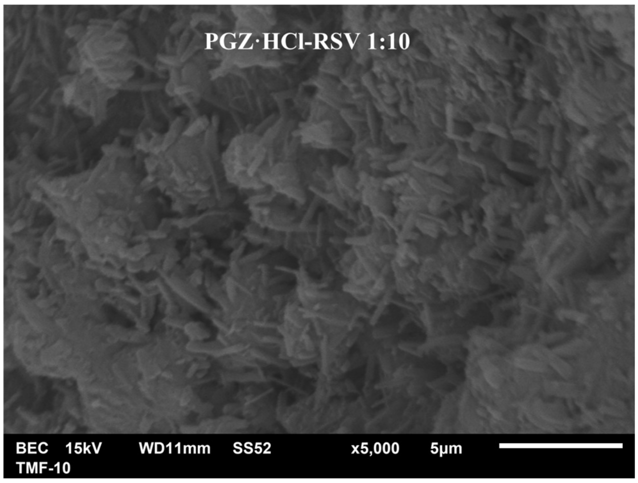
PGZ·HCl-RSV 1:10
BEC 15kV WD11mm SS52 x5,000 5µm
TMF-10

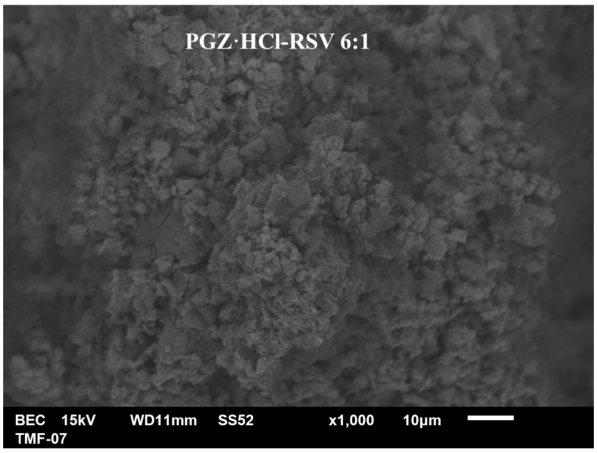
PGZ·HCl-RSV 6:1
BEC 15kV WD11mm SS52 x1,000 10µm
TMF-07

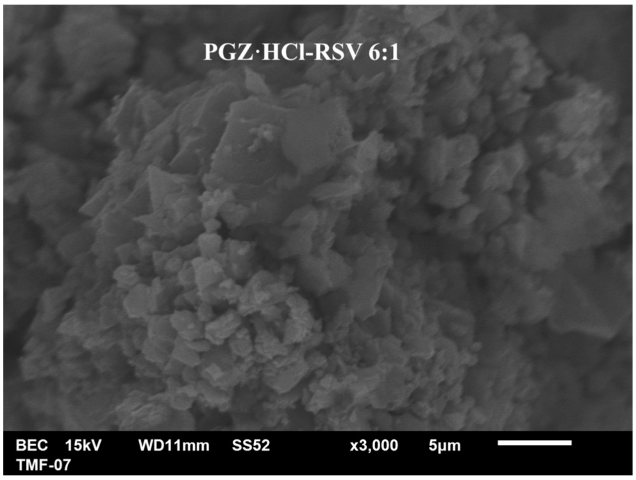
PGZ·HCl-RSV 6:1
BEC 15kV WD11mm SS52 x3,000 5µm
TMF-07

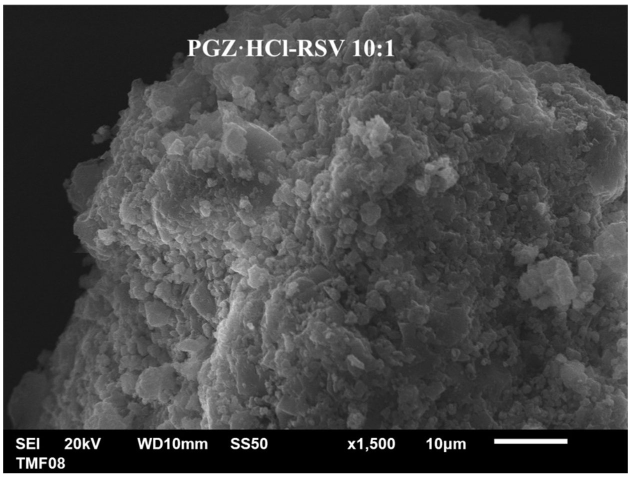

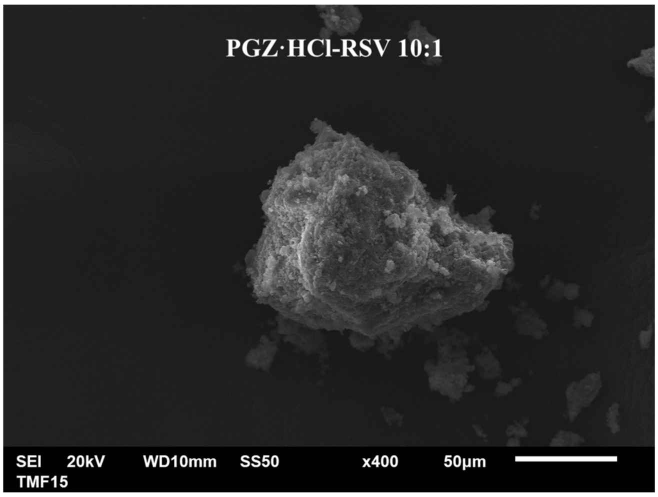

Figure S12. Grain morphology images of the solid forms (1:1; 1:6; 1:10; 6:1; and 10:1)
